# Remote-Sensing-Combined Haplotype Analysis Using Multi-Parental Advanced Generation Inter-Cross Lines Reveals Phenology QTLs for Canopy Height in Rice

**DOI:** 10.3389/fpls.2021.715184

**Published:** 2021-10-15

**Authors:** Daisuke Ogawa, Toshihiro Sakamoto, Hiroshi Tsunematsu, Noriko Kanno, Yasunori Nonoue, Jun-ichi Yonemaru

**Affiliations:** ^1^Institute of Crop Science, National Agricultural and Food Research Organization, Tsukuba, Japan; ^2^Institute for Agro-Environmental Sciences, National Agriculture and Food Research Organization, Tsukuba, Japan

**Keywords:** canopy height, GWAS, haplotype, high-throughput phenotyping, magic, phenology, QTL, UAV

## Abstract

High-throughput phenotyping systems with unmanned aerial vehicles (UAVs) enable observation of crop lines in the field. In this study, we show the ability of time-course monitoring of canopy height (CH) to identify quantitative trait loci (QTLs) and to characterise their pleiotropic effect on various traits. We generated a digital surface model from low-altitude UAV-captured colour digital images and investigated CH data of rice multi-parental advanced generation inter-cross (MAGIC) lines from tillering and heading to maturation. Genome-wide association studies (GWASs) using the CH data and haplotype information of the MAGIC lines revealed 11 QTLs for CH. Each QTL showed haplotype effects on different features of CH such as stage-specificity and constancy. Haplotype analysis revealed relationships at the QTL level between CH and, vegetation fraction and leaf colour [derived from UAV red–green–blue (RGB) data], and CH and yield-related traits. Noticeably, haplotypes with canopy lowering effects at *qCH1-4, qCH2*, and *qCH10-2* increased the ratio of panicle weight to leaf and stem weight, suggesting biomass allocation to grain yield or others through growth regulation of CH. Allele mining using gene information with eight founders of the MAGIC lines revealed the possibility that *qCH1-4* contains multiple alleles of *semi-dwarf 1* (*sd1*), the IR-8 allele of which significantly contributed to the “green revolution” in rice. This use of remote-sensing-derived phenotyping data into genetics using the MAGIC lines gives insight into how rice plants grow, develop, and produce grains in phenology and provides information on effective haplotypes for breeding with ideal plant architecture and grain yield.

## Introduction

Crops dramatically change during their cultivation in the field in terms of mass, morphology, and colours, and these changes are similar when grown in the same cultivation region and season. These phenological aspects in crops are possibly acquired through domestication (Gong, 2020; Lu et al., 2020) and are important to farmers in terms of efficient seasonal farming activities and maximisation of yields in a single harvest. How crops grow and develop differs depending on their genotypes, which often determine yield by regulating the transition from vegetative to the reproductive stage (Xue et al., 2008; Hill and Li, 2016). So far, to increase rice yields in breeding studies, genetic approaches using a population with natural variation and artificial mutation lines have largely focused on genes related to panicle number (PN), grain number per panicle, and grain weight (Xing and Zhang, 2010; Miura et al., 2011; Yin et al., 2021). On the other hand, how growth patterns during the cultivation term affect yield have not been examined in the field. One reason is that manual time-series measurement of crop growth is time-consuming and laborious. Another reason is that remote-sensing technology for observing crops has not been popular and familiar for breeding researchers. However, the advent of low-cost, user-friendly unmanned aerial vehicles (UAVs) is changing this situation.

Unmanned aerial vehicles can be used as non-destructive high-throughput phenotyping tools in the field and can facilitate the rapid observation of thousands of crop lines. A series of images covering a cultivation field are combined into an orthomosaic image using structure-from-motion/multi-view-stereo (SfM/MVS) software with high-accuracy ground control points (GCPs) (Jin et al., 2017; Weiss and Baret, 2017), enabling observation of a time-course of the changes in each crop line. Image data analysis can now be performed even by consumer-grade personal computers, and UAVs can be equipped with various sensors, such as digital red–green–blue (RGB), thermal, multispectral, and hyperspectral sensors (Gracia-Romero et al., 2019). From the image data derived from the sensors, specific phenotypes, such as vegetation fraction (VF), plant height, architecture, drought adaptability, and disease severity, have been evaluated (Gracia-Romero et al., 2017; Madec et al., 2017; Condorelli et al., 2018; Zhang et al., 2018; Chen et al., 2019; Hassan et al., 2019; Li et al., 2019; Marcial-Pablo et al., 2019; Ogawa et al., 2019; Wang et al., 2019b) and used for estimation of biomass and yield (Yue et al., 2017; Gong et al., 2018; Di Gennaro et al., 2019; Duan et al., 2019; Wang et al., 2019a).

One of the major issues in the use of UAVs in breeding studies is that the digital data deviate from the traditional traits related to yield, which are manually investigated using the conventional method using a ruler and scale. If the gaps between the image data and traditional trait data can be narrowed, it will accelerate the use of UAV imagery in the breeding study. In our previous study, we showed that VF calculated from UAV imagery is related to shoot dry weight during the vegetative stage in rice when observed in a population of multi-parental inter-mated lines [known as multi-parental advanced generation inter-cross (MAGIC) lines] named Japan-MAGIC (JAM) (Ogawa et al., 2021). A genome-wide association study (GWAS) using haplotype information in the JAM lines identified four quantitative trait loci (QTLs) for VF. Noticeably, the correlation between the VF and panicle weight (PW), grain yield trait was detected at the QTL level, suggesting that genetic analysis has the potential to make connections between high-throughput phenotyping data derived from UAV imagery and traditional yield trait data. This motivated us to examine another physical indicator, plant height, to reveal how vertical growth influences traits related to yield because VF represents growth only in two dimensions.

Plant height in crops is an important trait for plant architecture, affecting lodging tolerance and yield (Sakamoto and Matsuoka, 2004; Liu et al., 2018). Historically known as the “green revolution,” the introduction of semi-dwarf gene alleles resulted in remarkable increases in yields of wheat and rice (Peng et al., 1999; Sasaki et al., 2002). Plant height is regulated by many factors involved in the biosynthesis of and signal transduction by phytohormones, gibberellins (GA), brassinosteroids, and strigolactones (Salas Fernandez et al., 2009; Liu et al., 2018). Although the QTL analysis using genetically characterised populations and time-course plant height data has begun in wheat, maize, and rice (Tanger et al., 2017; Wang et al., 2019b; Lyra et al., 2020), the timing of the action of the genetic factors for plant height during the cultivation term and how they have an effect on plant architecture, biomass, and yield are scarcely known.

In this study, we focused on rice canopy height (CH) derived from UAV imagery. From 2 years of field experiments using the JAM lines, we showed that CH is a genetic trait. Genetic analysis using the JAM lines identified QTLs for CH (*qCH*), some of which have phenological features of growth stage specificity in their effects on CH. Haplotype analysis showed correlations of CH with other types of image data including VF and colour and with traits related to yield. Characterisation of these QTLs will facilitate the breeding of ideal rice varieties.

## Materials and Methods

### Cultivation of JAM Lines

The JAM population is derived from eight founders (Ogawa et al., 2018b): “Akidawara” (AK), “Bekogonomi” (BE), “Tachiaoba” (TC), “Mizuhochikara” (MI) (all *japonica*), “Suwon 258” (SU), “Takanari” (TK), “Hokuriku 193” (HO), and “Ruriaoba” (RU) (all *indica*). We cultivated 165 JAM lines (F_7_ in 2018 and F_8_ in 2019) without replication according to standard procedures at NARO in Tsukuba, Japan as described previously (Ogawa et al., 2021). In brief, seeds soaked in water at 28°C for 2 days were sown in trays filled with soil and incubated at 30°C in the dark for 2 days. Seedlings were grown in a paddy field in Kannondai, Tsukuba, for around a month, and then 33 seedlings per line were transplanted (11 plants 18 cm apart ×3 rows 30 cm apart, no replicates) into a nearby paddy field and grown for 5 months.

### UAV-Based Aerial Photography

Aerial images were taken in the same way as described previously (Ogawa et al., 2021). In brief, a Phantom 4 Pro UAV (P4P; DJI, Shenzhen, China) flew automatically above the field at 10 m altitudes. The flight path and image shooting setting were planned by DJI GS Pro software as follows: capture mode, time interval; speed, 1.0 m/s; front overlap ratio, 80%; side overlap ratio, 79%; gimbal pitch angle, −90°; image size, 3:2 (5,472 × 3,648 pixels); image format, JPG; white balance, cloudy; aperture, auto; shutter, auto; and exposure compensation value, −1 or 0. To set the focus, the P4P was manually raised to 10 m, the camera was focused automatically on a region of the canopy, and then the focus mode was changed to manual. On GCPs, we painted black and white markers at eight points on paved surfaces surrounding the field and precisely measured the latitude, longitude, and altitude of each point with a TCRP1205 surveyor (Leica, Heerbrugg, Switzerland).

### Generation of Orthomosaic Images and Digital Surface Model From the UAV Images

In Agisoft Metashape Professional v. 1.6.5 software (Agisoft, St. Petersburg, Russia), we generated an orthomosaic image from each set of aerial images using the following steps as described (Ogawa et al., 2019): (1) align photos (accuracy, high), (2) input GCPs, (3) build mesh (surface type, height field; source data, sparse cloud), (4) build digital elevation model (DEM; source data, sparse cloud), (5) calibrate colours (source data, model; calibrate white balance, no), and (6) build orthomosaic (surface DEM; blending mode, mosaic). A digital surface model (DSM) was also generated from the same dataset in the following steps: (1) align photos (accuracy, high), (2) input GCPs, (3) build dense cloud (accuracy, high), (4) build mesh (surface type, height field; source data, dense cloud), and (5) build DEM (source data, dense cloud). The orthomosaic images and the DSM images were analysed in ENVI v. 5.5 remote-sensing software (Harris Geospatial, Boulder, CO, USA). The map projection was converted to UTM zone 54N (WGS-84) with a 2-mm/pixel resolution. The converted image was rotated 66° clockwise to match the long-side direction of the field with the lateral direction of the final output image. Then the image was resized to a rectangle (28,000 × 14,000 pixels) including the field and the eight markers to minimise file size and thus processing time. The configuration of the computer used in this study is as follows: Intel(R) Core i7-6850-K CPU@3.8 GHz, 128 GB RAM, two NVIDIA GeForce GTX 1080Ti GPUs, and Windows 10 Pro, 64-bit operating system. It took 4 h for the whole process to create and analyse an orthomosaic image derived from about 600 images of a 30 m × 50 m field area. The workflow of the image capture and analysis are shown in Supplementary Figure 1.

### Quantification of CH

We made a DSM of the paddy field from a series of UAV RGB data with GCPs (Supplementary Figure 2) and estimated the CH by taking the difference in DSM between the day before transplanting and the observation date. We conducted a field survey to measure the CH of the 165 JAM lines by using a handy laser range finder (GLM 50 C, Robert Bosch, Gerlingen, Germany) and confirmed that the correlation coefficient between CH from the model and manual measurement was high (*r* = 0.87) (Supplementary Figure 3).

### Quantification of VF

The vegetation fraction was calculated as described previously (Ogawa et al., 2019). In brief, regions covering 3 × 3 plants of each JAM line in orthomosaic images were extracted, and RGB data were converted to the L^*^a^*^b^*^ colour space (León et al., 2006). The a^*^ data were used for auto-image thresholding by the Otsu method to create binary images for extracting plant regions, and the percentage of the number of pixels of the plant region to the total number of pixels was defined as the VF.

### Haplotype-Based GWAS

Haplotypes in the 165 JAM lines were estimated as in an Arabidopsis MAGIC population (Kover et al., 2009) from sequencing data of the eight founders and of 13,603 single-nucleotide polymorphisms (SNPs) determined by genotyping-by-sequencing analysis (Ogawa et al., 2018a,b). The haplotypes at each SNP were defined from the genotypes of the founders. Haplotype-based GWAS used haplotype information at each SNP and phenotype data in non-parametric ANOVA (Kruskal–Wallis rank-sum test) in the “kruskal.test” package of R software. The *p*-values obtained from the statistical analysis were used for the Manhattan plot. To identify *qCH* QTLs, we selected SNPs with a *p* < 1.0 × 10^−2^ in 2019 and 2018, and with the product of the two *p*-values is < 1.0 × 10^−5^. In the case of consecutive selected SNPs, the SNP with the lowest *p*-value defined the QTL unless the distance between them was more than 2 Mb. The effect of the haplotype on CH at each *qCH* QTL position was calculated using CH (Supplementary Data 1) and haplotype (Supplementary Data 2) data. We defined an averaged CH value per haplotype as a haplotype effect on CH. If the number of haplotypes was less than four (Supplementary Data 3), the data were not used for this study. We calculated the ratio of the average CH value for each haplotype to the average CH for all of the JAM lines. The haplotype effect was illustrated on scatter plots using R software.

### Manual Measurement of Traits Related to Yield

Culm length (CL) and panicle length (PL) were assessed on the longest culm in each plant and measured with a ruler from 10 days to a month after heading. At the time of the measurement, the PN was counted. For measurement of PW and stem and leaf weight (SLW), shoots of mature plants were dried for over a month in a drying room and cut 3 cm below the panicle base to separate the parts (Ogawa et al., 2021). The total weight (TW) was calculated as PW + SLW. CL, PL, PN, PW, SLW, and TW values for each JAM line are averages of five plants. SDW was measured as described previously (Ogawa et al., 2021). All of the abbreviations in this manuscript are listed in Table 1.

**Table 1:** List of abbreviations.

**Abbreviation**	**Explanation**
AK	Akidawara (one of the founders of JAM lines)
BE	Bekogonomi (one of the founders of JAM lines)
CH	Canopy height
CL	Culm length
DAT	Days after transplanting to the field
DEM	Digital elevation model
DSM	Digital surface mode
GA	Gibberellin
GCP	Ground control point
GWAS	Genome-wide association studies
HO	Hokuriku193 (one of the founders of JAM lines)
JAM	Japan-MAGIC
MAGIC	Multi-parental advanced generation inter-cross
MI	Mizuhochikara (one of the founders of JAM lines)
PL	Panicle length
PN	Panicle number
PW	Panicle weight
*qCH*	QTL for CH
QTL	Quantitative trait loci
*qVF*	QTL for VF
RGB	Red-green-blue
RU	Ruriaoba (one of the founders of JAM lines)
*sd1*	*Semi-dwarf 1*
SDW	Shoot dry weight
SfM/MVS	Structure-from-motion/multi-view-stereo
SLW	Stem and leaf weight
SNP	Single nucleotide polymorphism
SU	Suwon258 (one of the founders of JAM lines)
TC	Tachiaoba (one of the founders of JAM lines)
TK	Takanari (one of the founders of JAM lines)
TW	Total weight (PW + SLW)
UAV	Unmanned aerial vehicles
VF	Vegetation fraction

## Results

### Detection of QTLs for CH

We observed how CH in the JAM lines changed during the cultivation term using UAV imagery. The average CH in the JAM lines increased for 83 days after transplanting to the field (DAT) and decreased moderately after that (Supplementary Figure 4A). This appears to be related to the vertical growth of rice plants until tillering and heading, followed by drooping of stems and panicles during maturation. Wide phenotypic variation was observed in the JAM lines at 83 DAT in both years. Positive correlations were detected in the timing after transplanting of six developmental stages (two tillering, heading, and maturation stages) in the 2-year data (Supplementary Figure 4B), indicating that the CH is a heritable trait.

**Figure 1:**
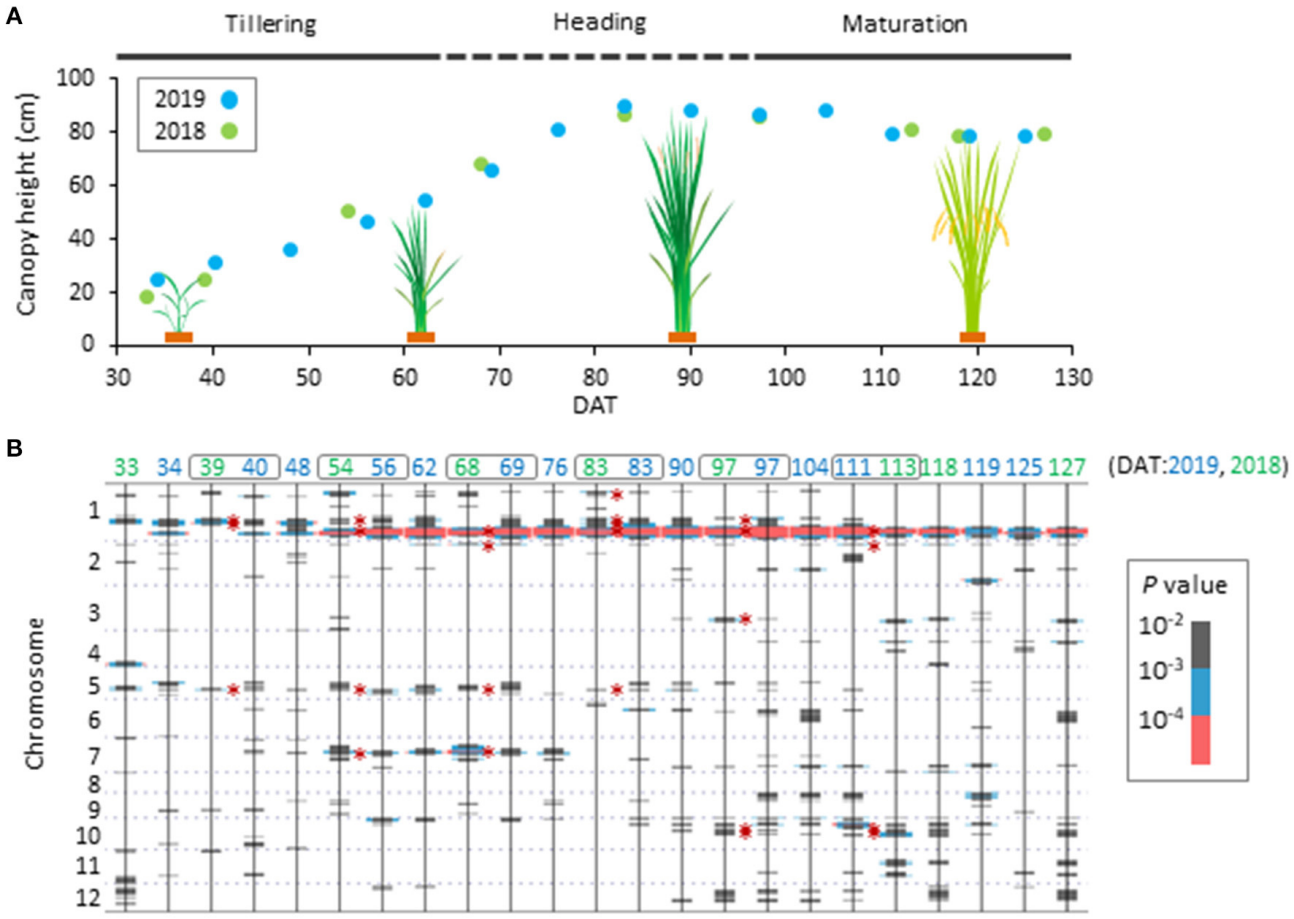
Time-course pattern of QTL appearance in canopy height of JAM lines. **(A)** Scheme of phenological change of canopy height (CH) of the JAM lines. Average values of canopy height (CH) of the 165 JAM lines were plotted. There are tillering, heading and maturation stages in the developmental process. **(B)** Summary of haplotype-based genome wide association study on CH using the 165 JAM lines. Results from data collected in 2019 and 2018 are shown together arranged in ascending order of DAT from left to right. Bars in grey, blue and pink were added at positions where *P*-values are lower than 10^−2^. Six time points of DAT indicated by grey boxes were focused on in this study. Defined QTL regions are marked with asterisks.

To determine which genetic factors are involved in controlling CH, we performed a haplotype-based GWAS using CH data and haplotype information on 13,603 SNPs of the 165 JAM lines. As a result, we found 11 QTLs (Figure 1, Table 2, Supplementary Figure 5), and named them *qCH1-1, qCH1-2, qCH1-3, qCH1-4, qCH2, qCH3, qCH5-1, qCH5-2, qCH7, qCH10-1*, and *qCH10-2*. The time points when the QTLs were detected were different among the QTLs; *qCH1* and *qCH5-1*, and *qCH3, qCH10-1*, and *qCH10-2* were heading and maturation stage-specific, respectively. *qCH1-2* and *qCH1-4* were detected at the tillering, heading, and maturation stages.

**Table 2:** Quantitative trait loci (QTLs) for canopy height (CH) shown in Figure 1.

			**DAT (2019, 2018)**						
			**40, 39^†^**	**56, 54^†^**	**69, 68^†^**	**83, 83^†^**	**97, 97^†^**	**111, 113^†^**	
**Name of QTL**	**Chr**	**Pos**	**Tillering**		**Heading**		**Maturation**		**Candidate gene or QTL**
*qCH1-1*	1	5,774,227	–	–	–	+	–	–	–
*qCH1-2*	1	29,262,844 – 29,617,334	+	+	–	+	+	–	*qVF1*
*qCH1-3*	1	32,039,637	+	–	–	+	–	–	
*qCH1-4*	1	38,560,504 – 39,210,519	–	+	+	+	+	+	*Sd1*
*qCH2*	2	3,370,339 – 3,456,275	–	–	+	–	–	+	–
*qCH3*	3	28,908,224	–	–	–	–	+	–	–
*qCH5-1*	5	21,564,904	–	–	+	–	–	–	–
*qCH5-2*	5	24,493,293	+	+	–	+	–	–	–
*qCH7*	7	15,513,069	–	+	+	–	–	–	–
*qCH10-1*	10	87,974	–	–	–	–	+	+	–
*qCH10-2*	10	4,445,470 – 4,699,327	–	–	–	–	+	+	–

† *DATs in 2019 (left) and in 2018 (right) are shown*.

**Figure 2:**
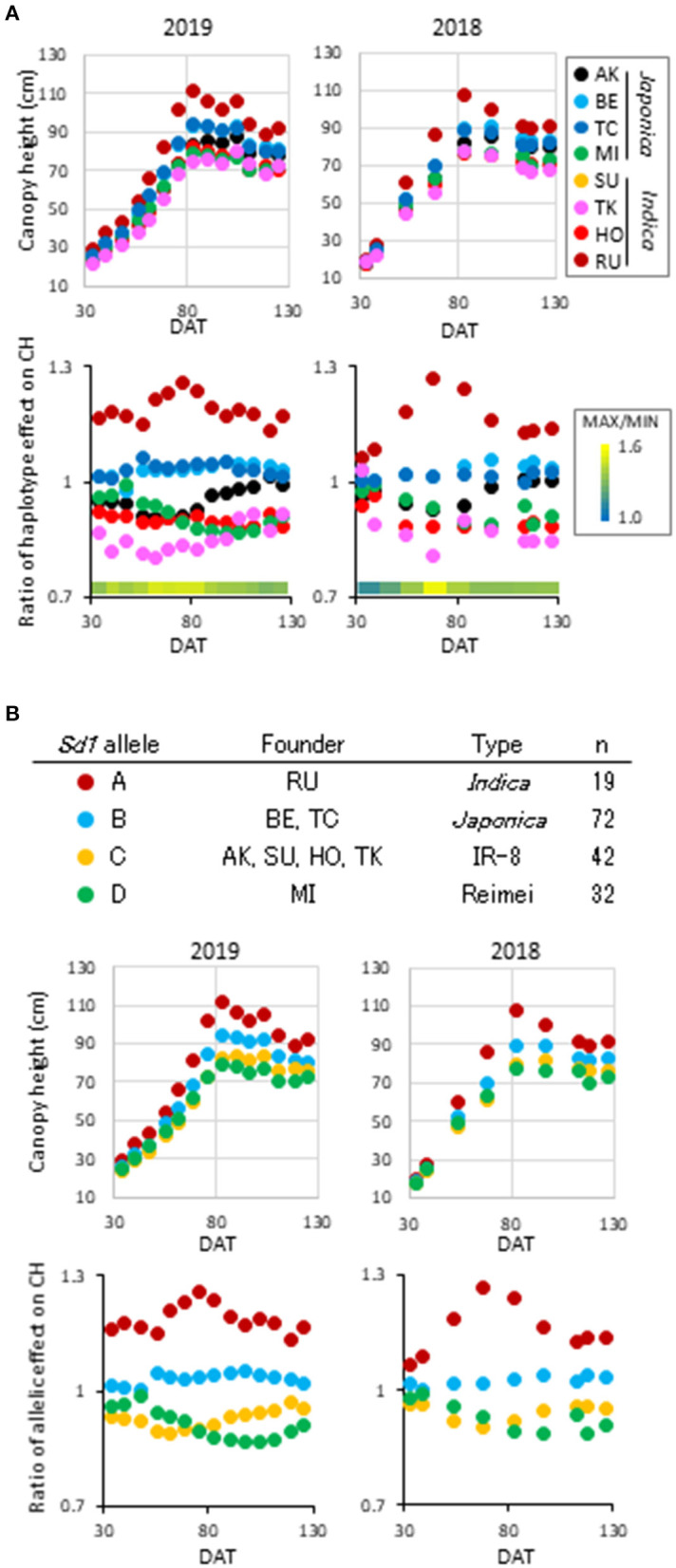
Haplotype effect on canopy height at *qCH1-4*. **(A)** Time-course pattern of haplotype effect on CH at *qCH1-4*. Average phenotypic values of the eight haplotypes (top) and ratio of the haplotype effect to average of the JAM lines (bottom) at each DAT in 2019 (left) and 2018 (right) are plotted. **(B)** Time-course pattern of *Sd1* allelic effect on CH at *qCH1-4*. Four *Sd1* alleles were included in the eight founders (top). Average phenotypic values of the four alleles (middle) and ratio of the allelic effect to average of the JAM lines (bottom) at each DAT in 2019 (left) and 2018 (right) are plotted.

Among the *qCH* QTLs, we first focused on *qCH1-4*, where the most remarkable peak was detected (Figure 1, Supplementary Figure 5) close to the *semi-dwarf 1* (*sd1*) gene. The CH in lines possessing each of the haplotypes at *qCH1-4* on CH was most different at 83 DAT in 2019 and 68 DAT in 2018 (Figure 2A). There are four alleles of the *Sd1* gene in the eight founders of the JAM lines, as described previously (Ogawa et al., 2018b). When the lines were classified by their *Sd1* gene allele based on the haplotype information, the CH of three classes (c: allele A, b: allele B, a: allele C and D) was statistically differentiated from 56 to 111 DAT in 2019 and from 68 to 97 DAT in 2018 (Figure 2B, Supplementary Figure 6), which is during the late tillering stage and early maturation stage. This *Sd1* allelic pattern is consistent with the allele function presumed from data of CL (Ogawa et al., 2018b), indicating that *Sd1* is a candidate gene for *qCH1-4*.

**Figure 3:**
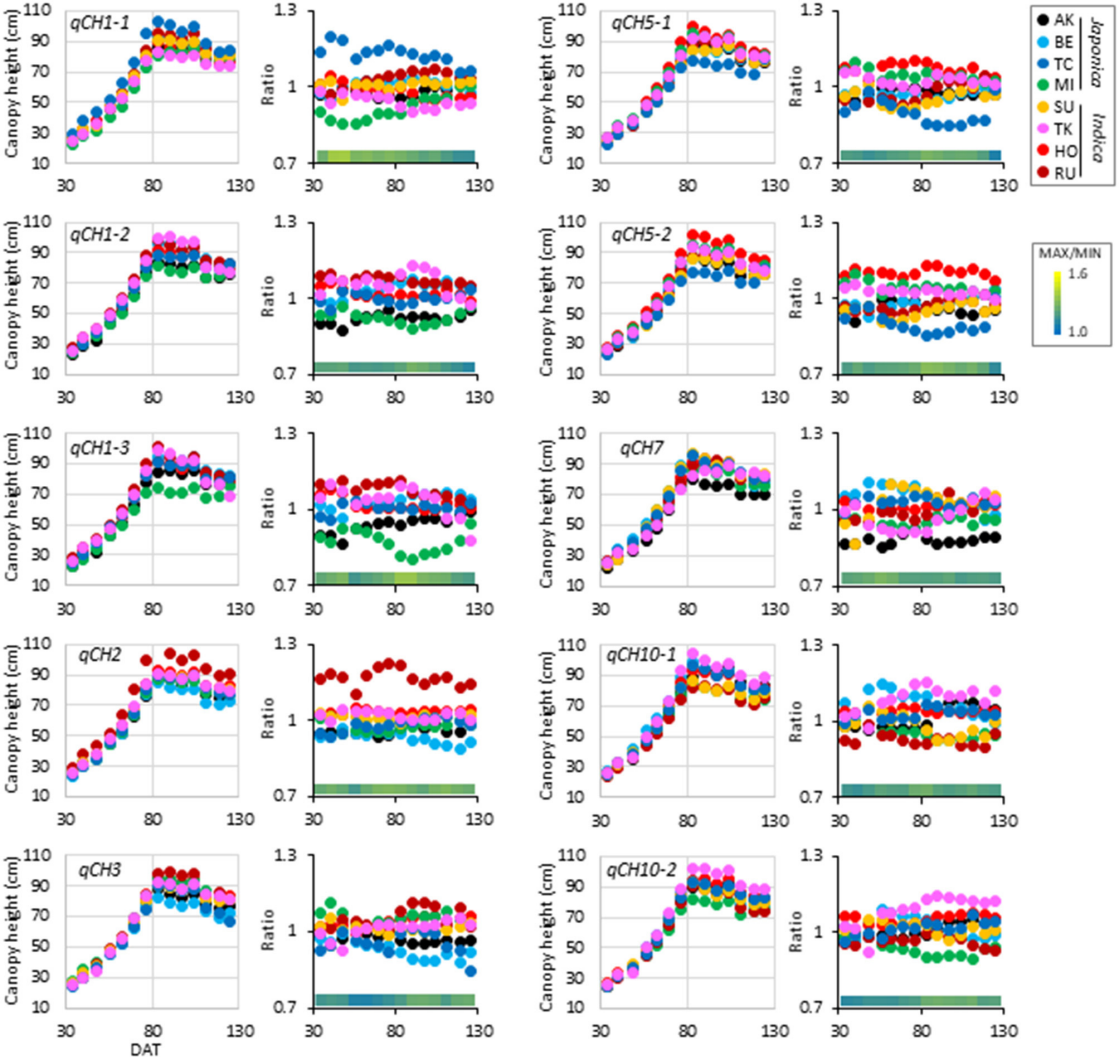
Time-course pattern of haplotype effect on CH at *qCH* QTLs except for *qCH1-4*. Average of haplotype effect on CH (left) and ratio of the haplotype effect to the average values in the JAM lines (right) at each DAT in 2019 were plotted.

The time-course pattern of haplotype effect on CH was different among the *qCH* QTLs (Figures 2A, 3, Supplementary Figure 7, Supplementary Data 4). To characterise the QTLs, hierarchical clustering analysis using correlation coefficient *r*-values of all CH data in 2019 and 2018 was performed (Supplementary Figures 8, 9). Pairs of QTLs close in chromosome position were located in the same clusters, such as *qCH5-1* and *qCH5-2, qCH1-2* and *qCH1-3*, and *qCH10-1* and *qCH10-2* because their pattern of haplotypes is similar between the two QTLs (Supplementary Data 2). Although *qCH1-4* and *qCH2* are on different chromosomes, they were located in the same cluster, where the haplotype effect on CH tended to be relatively stable through rice development and cultivation years (Figures 2A, 3, Supplementary Figure 7). Similar to *qCH1-4*, haplotype [RU] at *qCH2* had a greater ability to increase CH. When the time-course haplotype data on CH was modelled using a quadratic curve (Supplementary Figure 10), the coefficients showing the sharpness of curve for [RU] at *qCH1-4* and *qCH2* were remarkably lower than for the other haplotypes.

### Effect of *qCH* on Vegetation Fraction

*qCH1-2* was close to the position of *qVF1* (Ogawa et al., 2021), which is a QTL for VF, detecting using images as shown in Supplementary Figure 11. To examine the relationship between CH and VF at all *qCH* QTLs including *qCH1-2*, we carried out correlation analysis at the haplotype level. The haplotype effects on CH and VF were highly correlated at *qCH1-1, qCH1-2*, and *qCH1-3* (Supplementary Figure 12A), indicating that these QTLs contribute to both lateral and vertical growth. At *qCH1-2* and *qCH1-3*, the *indica* haplotypes [RU, HO, and TK] caused increased CH and VF relative to the *japonica* haplotypes [AK, BE, TC, and MI] (Supplementary Figure 12B), and CH was also related to shoot dry weight (SDW) (Supplementary Figure 12C).

### Effect of *qCH* on Leaf Colour

It has been reported that stem and leaf elongation and leaves turning pale green are induced by the effect of plant hormone gibberellin (GA) in *Arabidopsis* (Jacobsen and Olszewski, 1993; Olszewski et al., 2002). It is well-known that GA plays a role in controlling leaf length and CL in rice (Sasaki et al., 2002; Liu et al., 2018). This motivated us to examine if CH is correlated with leaf colour from tillering to maturation. We isolated the leaf region from orthomosaic images and obtained a^*^ data from RGB data (León et al., 2006). We used minus a^*^ (–a^*^) data as an indicator of leaf colour, which ranges from red (−128) to green (+128). The average –a^*^ in the 165 JAM lines increased until 69 DAT in 2019 and until 83 DAT in 2018 during the early heading stage and then decreased during maturation (Supplementary Figure 13A). The –a^*^ data at the selected six time-points were similar between 2019 and 2018 (Supplementary Figure 13B). We expected a negative correlation between CH and –a^*^ (green colour level of leaves) in the JAM lines like the GA response in *Arabidopsis*, but a simple negative correlation was not observed (Supplementary Figure 14).

**Figure 4:**
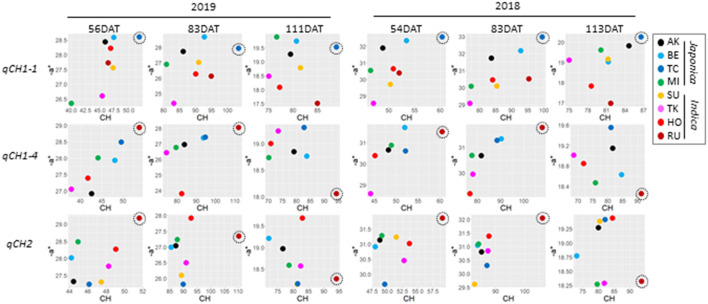
Difference of the relationship between CH and –a* data at *qCH* QTLs. Plots of the correlation of haplotype effect between CH and –a* at *qCH1-1* (top), *qCH1-4* (middle) and *qCH2* (bottom) in 2019 (left) and 2018 (right). Haplotype [TC] at *qCH1-1*, haplotype [RU] at *qCH1-4*, and haplotype [RU] at *qCH2* were focused on as remarkable haplotypes for CH, and are indicated by dotted circles.

At the QTL level, the relationship between CH and –a^*^ depended on the *qCH* QTLs and the time-point (Supplementary Figure 14). Correlation values between haplotype effects on CH and –a^*^ dramatically changed from 97 to 111 DAT in 2019 and from 83 to 113 DAT in 2018 during maturation at several *qCH* QTLs. To understand these changes, we examined haplotype effects on CH and –a^*^ in detail. We focused on the effects of haplotype (TC) at *qCH1-1*, haplotype (RU) at *qCH1-4*, and haplotype (RU) at *qCH2*, which have a strong ability to increase CH. The effect of haplotype (TC) at *qCH1-1* was kept on CH and –a^*^ at a high level until 111 DAT in 2019 and 113 DAT in 2018, but the other two haplotypes caused sudden drops in –a^*^ (Figure 4). This indicates that specific haplotypes can lead to rapid leaf senescence during maturation. Considering that *Sd1* encoding GA 20-oxidase is a candidate of *qCH1-4*, involvement of GA in leaf senescence is presumed.

### Effect of *qCH* QTLs on Traits Related to Yield

To clarify relationships between CH and traits related to yield, we first examined if CH contributes to traits including CL, PL, and PN. CH and CL in 165 JAM lines were highly correlated at 83 and 97 DAT in 2019 and 2018, but not after that, possibly because plant architecture including panicle and leaf positions drastically changed during maturation (Supplementary Figure 15A). Consistent with this, the patterns of haplotype effects on CH were highly similar to those on CL at all *qCH* QTLs (Supplementary Figures 15B, 16). The relationship between CH and PL was positive in the JAM lines in 2019 and 2018, but the relationship between CH and PN was negative (Supplementary Figure 15B). At the QTL level, the pattern of the haplotype effect on CH was positively related to that on PL clearly at *qCH1-1, qCH2*, and *qCH7* in 2019 and 2018 (Supplementary Figure 17A) and was negatively done to that on PN at *qCH1-4* and *qCH7* (Supplementary Figure 17B).

**Figure 5:**
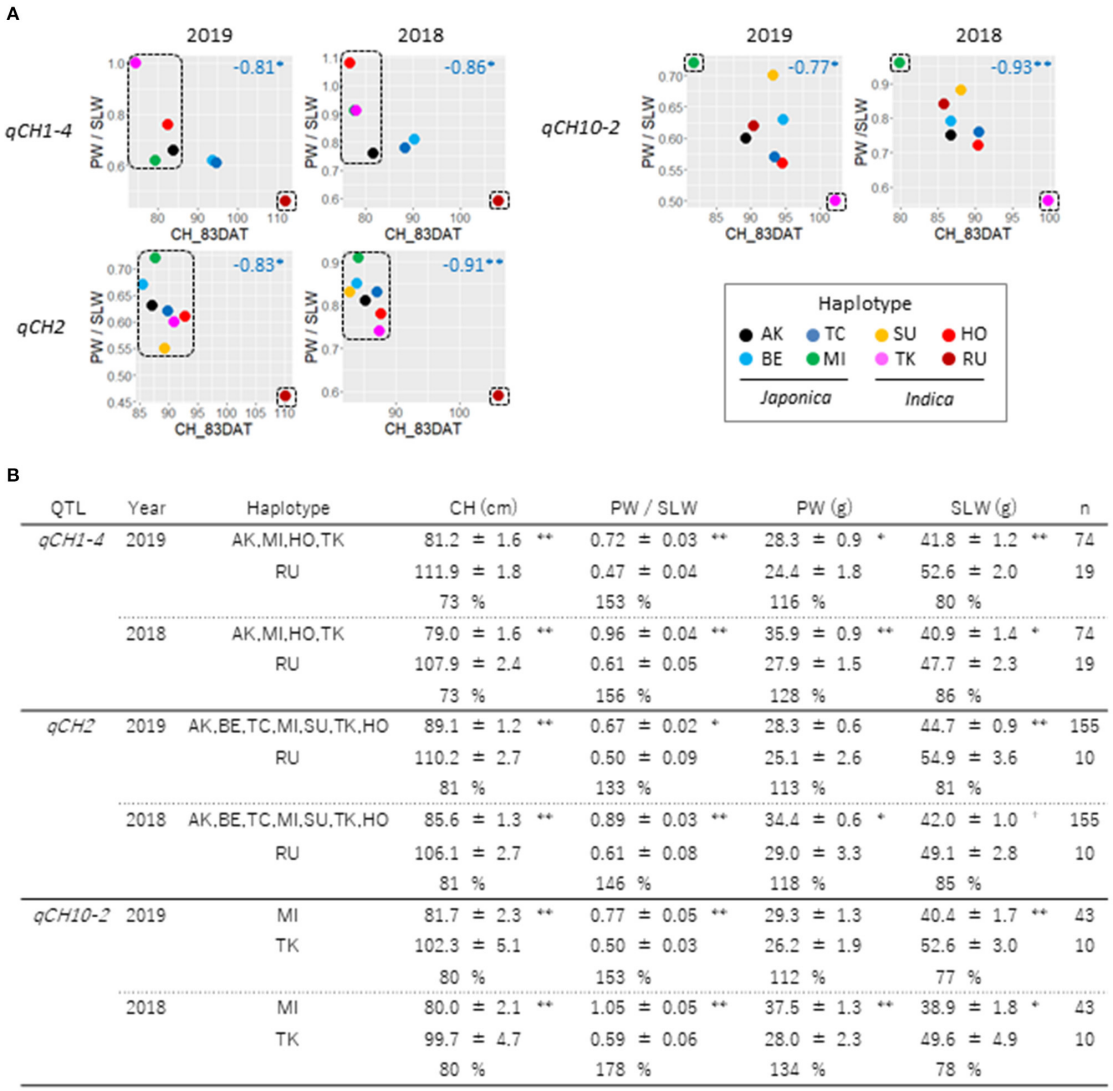
Relationships between CH and ratio of PW to SLW at *qCH* QTLs. **(A)** Haplotype effects on CH at 83DAT and ratio of PW to SLW in 2019 (left) and 2018 (right) at *qCH1-4, qCH2* and *qCH10-2* were plotted. Numbers in blue indicate Pearson's *r*. Asterisks indicate significant correlations (Pairwise two-sided, **P < 0.01, *P < 0.05). **(B)** Comparison of haplotype effects on CH, PW/SLW, PW and SLW at *qCH1-4, qCH2* and *qCH10-2* were shown. Data on haplotypes enclosed by dotted lines in **(A)** at the three QTLs were examined. Mean ± standard error of phenotypes and ratio of phenotype data in top haplotype class to that in bottom haplotype class at each QTL were shown (student *t*-test: **P < 0.01, *P < 0.05, ^+^*P* < 0.1).

We next examined if CH is associated with PW, SLW, and the TW. CH data tended to be weakly correlated to SLW and TW in 2019 and 2018, but not to PW (Supplementary Figure 15A). At the QTL level, the relationship of the haplotype effects on CH and TW was not robust in 2019 and 2018 (Supplementary Figure 15B). Correlation of haplotype effects on CH and SLW was observed at *qCH1-1, qCH1-2, qCH1-4, qCH2*, and *qCH10-2* in 2019 and 2018 although the time-points when the correlation was detected depending on the QTL. Intriguingly, among the *qCH* QTLs, a negative relationship between CH and PW was often found at *qCH1-4, qCH2*, and *qCH10-2*. When the ratio of PW to SLW was examined at the three QTLs, CH was negatively related to the ratio (Figure 5A). QTL haplotypes that reduced CH led to significantly increased PW and decreased SLW in contrast to those with the effect of increasing CH, at least once in the 2 years' experiments (Figure 5B). These results suggest regulation of the balance between PW and SLW by the QTLs for CH.

## Discussion

Traditional crop breeding research has focused mainly on traits that can be obtained in a single destructive inspection. This approach missed information about the timing and significance of changes in growth and development. Remote-sensing technology has the potential to compensate (Sakamoto et al., 2012; Sakamoto, 2018). In this study, we developed a CH measurement method using UAV imagery with rice cultivated in a paddy field and performed GWAS using time-course CH data and haplotype information in JAM lines. Due to the opportunity of multiple observations, we were able to identify 11 *qCH* QTLs, which were robust in the 2 years of the experiments. By analysing haplotype effects on CH, we revealed significant correlations of the haplotype data in 2019 and 2018 at all QTLs, and the persistence and transience of different haplotype functions in vertical growth showed differences in phenology among QTLs.

**Figure 6:**
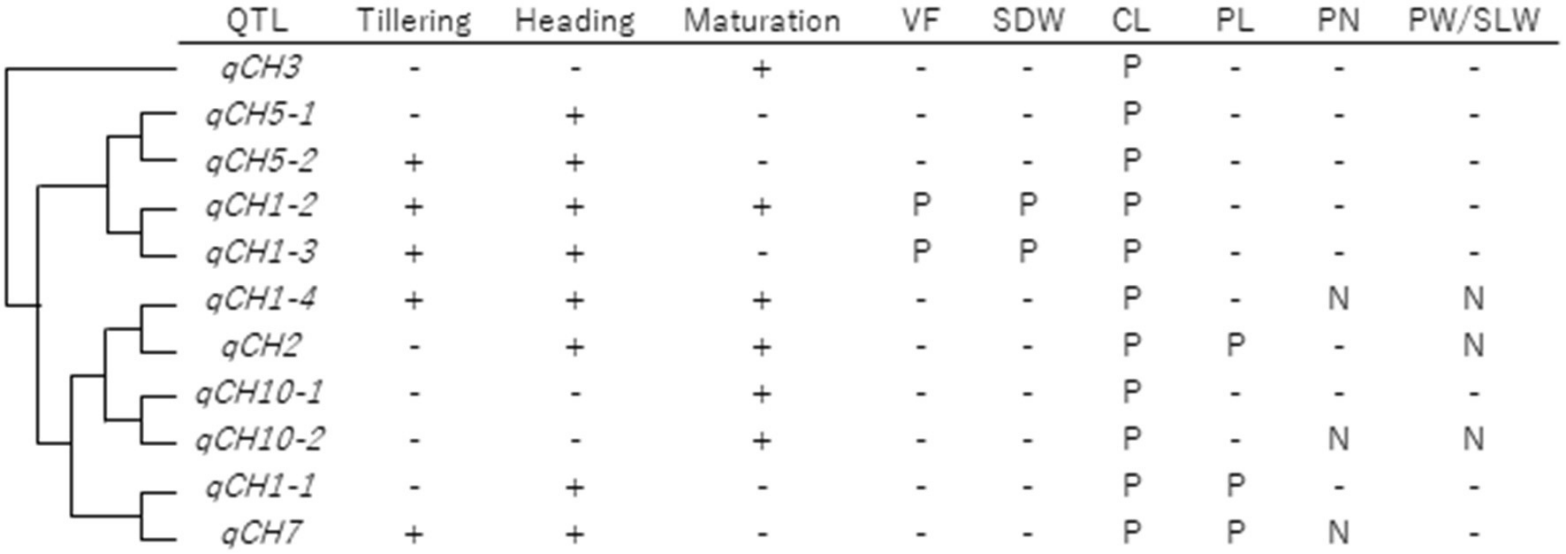
Summary of *qCH* QTLs. *qCH* QTLs were aligned in order of hierarchical clustering in terms of haplotype effect on CH in 2019 and 2018, shown in Supplementary Figure 9. Each QTL was detected in the three stages (tillering, heading, and maturation) and the positive (P) or negative (N) relationships of haplotype effects between CH and VF, SDW, CL, PL, PN, and PW/SLW were shown. In terms of VF and SDW, results shown in Supplementary Figure 12 were summarised. In terms of CL, PL, and PN, P or N was labelled when significant correlation (*P* < 0.05) was detected a total of twice or more among the six time points in 2019 and 2018. In terms of PW/SLW, results shown in Figure 5B were summarised.

As summarised in Figure 6, haplotype analysis using JAM lines revealed; (1) correlation between haplotype effects on CH and CL at all QTLs, (2) correlation between haplotype effects on CH and PL at *qCH1-1, qCH2*, and *qCH7*, and (3) negative correlation between haplotype effects on CH and PN at *qCH1-4* and *qCH7*. These results indicate differing pleiotropic effects of *qCH* QTLs on traits related to yield, except for CL. Furthermore, the negative correlation of haplotype effects on CH and the ratio of PW to SLW was detected at *qCH1-4, qCH2*, and *qCH10-2*, all of which function at least during the maturation stage. This indicates that CH contributes to increasing the final number of leaves and stems instead of grains in panicles under the control of haplotypes at *qCH1-4, qCH2*, and *qCH10-2*, implying the influence of phenological CH regulation on the allocation of photosynthetic products to leaves and stems or grains.

We also attempted to examine the relationship between CH and data derived from UAV imagery including VF and –a^*^, leaf colour. Haplotype analysis in terms of VF revealed a connection between lateral and vertical growth at *qCH1-2* and *qCH1-3*, which are close to *qVF1* (Ogawa et al., 2021). In the case of –a^*^ data analysis, rapid leaf senescence was observed for haplotype (RU) of *qCH1-4* and *qCH2*, which has a strong ability to increase CH. PW in JAM lines with this haplotype was lower than that in the other haplotypes, implying some relationship among yield, senescence, and timing, and intensity of shoot growth during the cultivation term. In Japan, only the cultivar RU among the eight founders of the JAM lines is cultivated for whole crop silage. The haplotype (RU) at *qCH1-4* and *qCH2* might play a central role to increase the amount of leaf and stem relative to grains.

Our haplotype approach detected *qCH1-4* as the most remarkable QTL for CH, whose candidate is the *Sd1* gene. *qCH1-4* affected the balance between the amount of leaf and stem and yield in this genetic study, showing the effectiveness of the green revolution with the *Sd1* gene in the JAM lines. In breeding, it is said that the overuse of *Sd1* in rice cultivars runs the risk of lowing genetic diversity (Liu et al., 2018). To avoid this risk, haplotypes at *qCH2* and *qCH10-2* with effects that lower CH may be options for the production of new semi-dwarf cultivars because they also induce an increase of yield like *qCH1-4*.

In the method of CH measurement, it took 30 min for capturing RGB images of 30 m × 50 m field area by using UAV and 4 h for image analysis. The method enabled the measurement of CH with high accuracy without any laborious steps, demonstrating an effective way to examine CH of rice lines grown in the field. We used the Agisoft MetaShape Professional v. 1.6.5 software to create orthomosaic images and ENVI software (Harris Geospatial, Boulder, CO, USA) to analyse phenotype data, which we have also been used for the analysis of VF and plant architecture in our previous works (Ogawa et al., 2019, 2021). We believe that both of the software is very useful for high-throughput phenotyping using UAVs because it is user-friendly and keeping updated. As alternative SfM/MVS software, Pix4D is known to create orthomosaic images for investigating phenotypes of crops (Zhang et al., 2018; Chen et al., 2019; Hassan et al., 2019; Li et al., 2019, Marcial-Pablo et al., 2019).

In this study, we showed correlations and trade-off relationships between CH and the other traits at the QTL level. It is suggested that QTLs for CH are key factors for plant architecture, biomass, and yield. This study was accomplished by the integration of the remote-sensing technology and genetics using our rice MAGIC population. Our methodology can generate more than 100,000 (8 haplotypes × 13,615 SNPs) haplotype patterns for each time-course of phenotype data in a 1-year experiment. If the number of SNPs is increased and the experiments continue, haplotype data with phenotypic information increase. Haplotype pattern analysis using recently developed massive data analysis methods, such as machine learning, will uncover further relationships between traits useful for the production of ideal varieties. This haplotype approach using a genetically characterised population can be applied to other high-throughput phenotyping data and other crops. We believe such trials will advance digital data-driven breeding.

## Data Availability Statement

The raw data supporting the conclusions of this article will be made available by the authors, without undue reservation.

## Author Contributions

DO and J-iY conceptualised the research. DO, TS, HT, NK, YN, and J-iY performed the investigations. DO, TS, and J-iY developed the methodology and performed the formal analysis. DO, NK, and YN provided the resources. DO, TS, HT, and J-iY performed data curation. DO, HT, and J-iY helped with funding acquisition. DO provided project administration and wrote the manuscript. J-iY helped with writing the original draft of the manuscript. TS and J-iY reviewed and edited the manuscript. All authors contributed to the article and approved the submitted version.

## Funding

This study was supported by grants from the Ministry of Agriculture, Forestry and Fisheries of Japan [Smart-breeding system for Innovative Agriculture (Grant Number: BAC1003)], and from the Science and Technology Research Promotion Program for Agriculture, Forestry, Fisheries and Food Industry (Grant Number: 27007B).

## Conflict of Interest

The authors declare that the research was conducted in the absence of any commercial or financial relationships that could be construed as a potential conflict of interest.

## Publisher's Note

All claims expressed in this article are solely those of the authors and do not necessarily represent those of their affiliated organizations, or those of the publisher, the editors and the reviewers. Any product that may be evaluated in this article, or claim that may be made by its manufacturer, is not guaranteed or endorsed by the publisher.
