# Supplementary material for: Remote-Sensing-Combined Haplotype Analysis Using Multi-Parental Advanced Generation Inter-Cross Lines Reveals Phenology QTLs for Canopy Height in Rice
Source: Front Plant Sci. 2021 Oct 15;12:715184. doi: 10.3389/fpls.2021.715184 (PMC8553969; doi:10.3389/fpls.2021.715184)
Supplement: Supplementary file 1 [file Presentation_1.pdf]

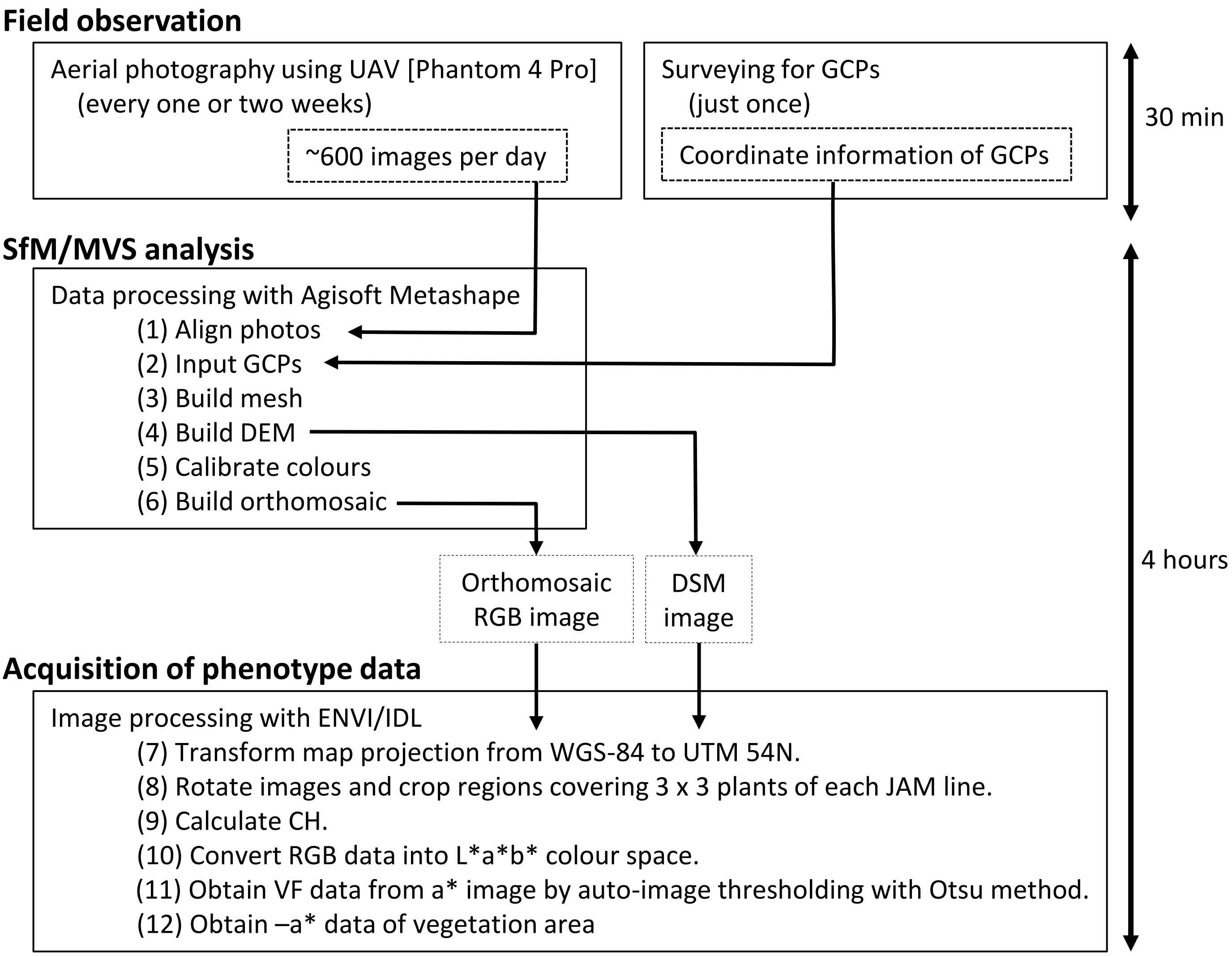

Supplementary Figure 1. Workflow of image capturing and analysis in this study. A series of RGB images of the rice field captured with UAV was processed with Agisoft Metashape and ENVI/IDL software to obtain CH, VF and –a* data. The processing time for the field observation and image analysis to investigate the phenotypes was shown on the right side.

Before cultivation
May 20th, 2019

40 DAT
July 1st, 2019

56 DAT
July 17th, 2019

69 DAT
July 30th, 2019

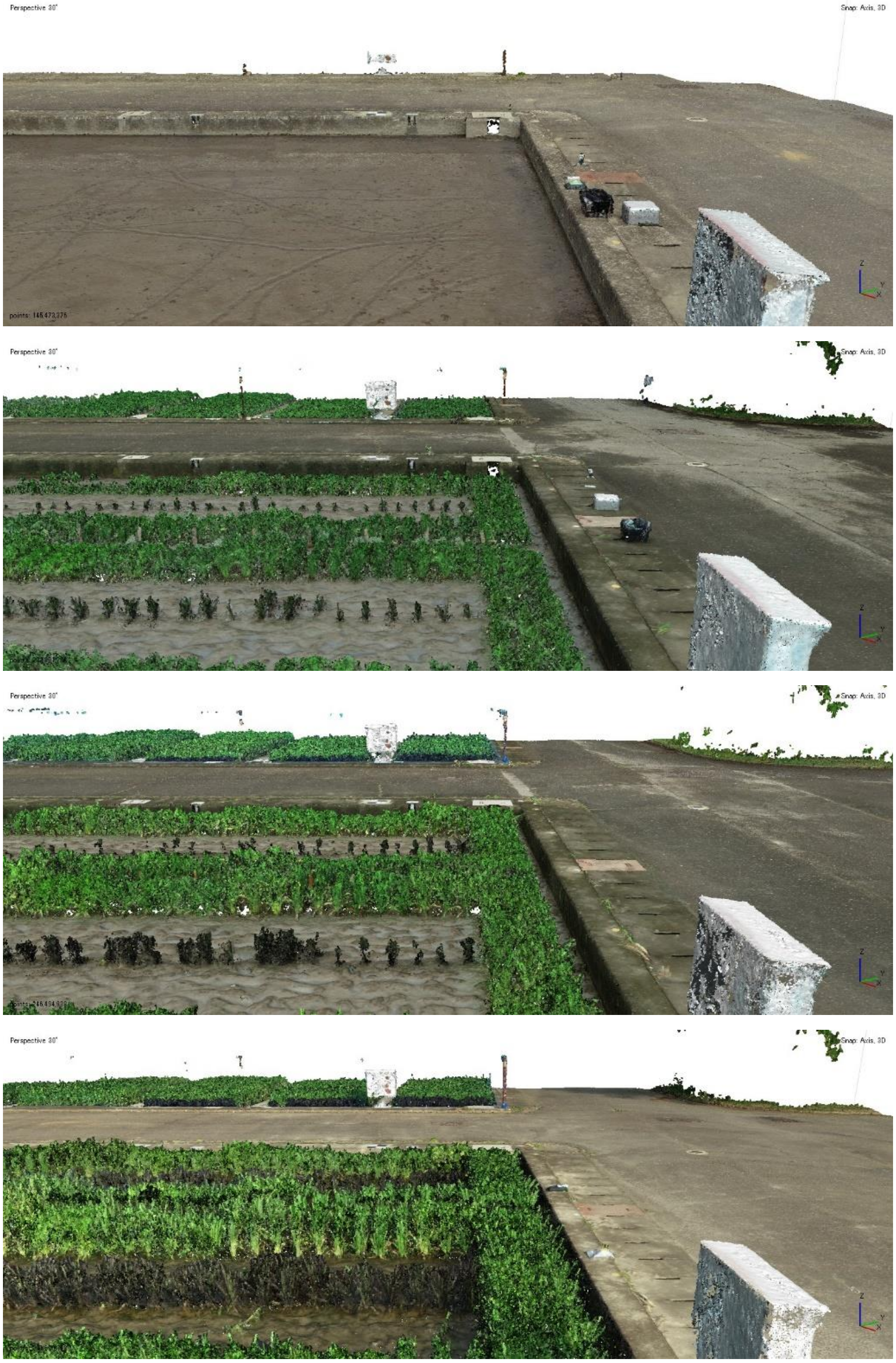

Supplementary Figure 2. Dense cloud data for constructing digital surface model (DSM)
The images were constructed from aerial photos taken from before cultivation to 69 DAT.

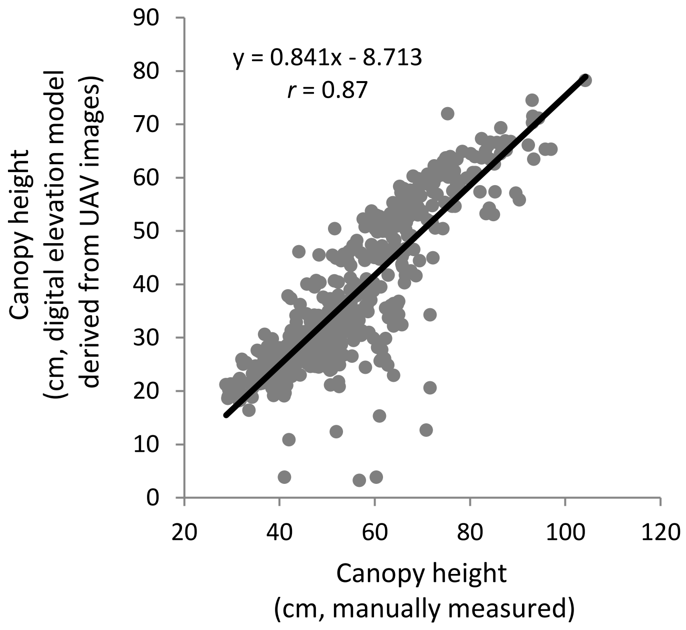

Supplementary Figure 3. Comparison of manually measured canopy height with canopy height calculated from digital surface model (DSM). Manually measured CH data and DEM-based CH data were plotted. The measurement was performed at 34, 40 and 62 DAT on the 165 JAM lines.

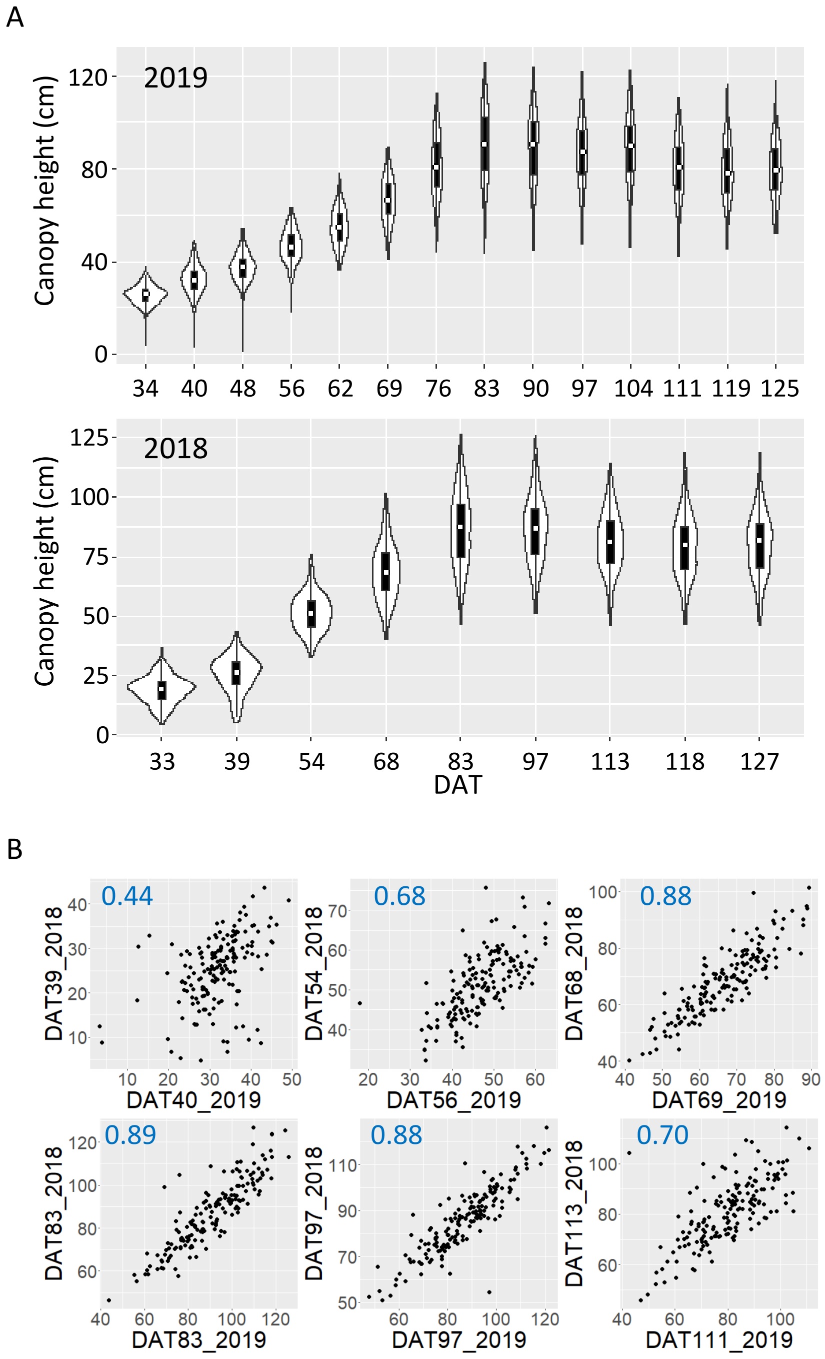

Supplementary Figure 4. Features of CH data in JAM lines.
(A) Violin plots of CH data of the 165 JAM lines in 2019 (top) and 2018 (bottom). (B) Relationship between data of CH in 2019 and 2018 at 6 DAT time points. Numbers in blue indicate Pearson's *r*.

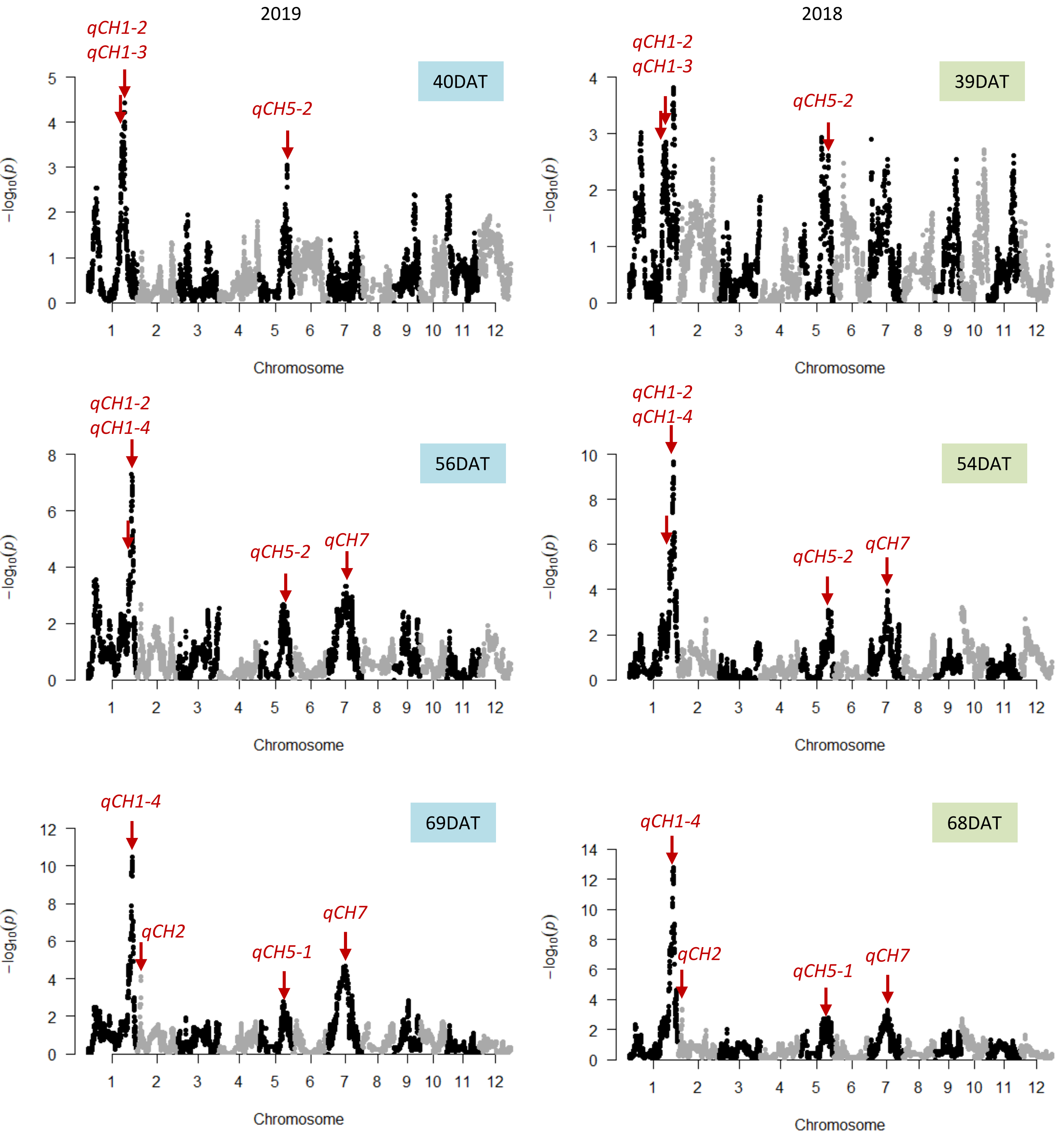

Supplementary Figure 5. GWAS of CH using JAM lines.
Manhattan plot of GWAS of CH at six points in 2019 (left) and 2018 (right) using haplotype data of 13,603 SNPs. Arrows indicate QTLs.

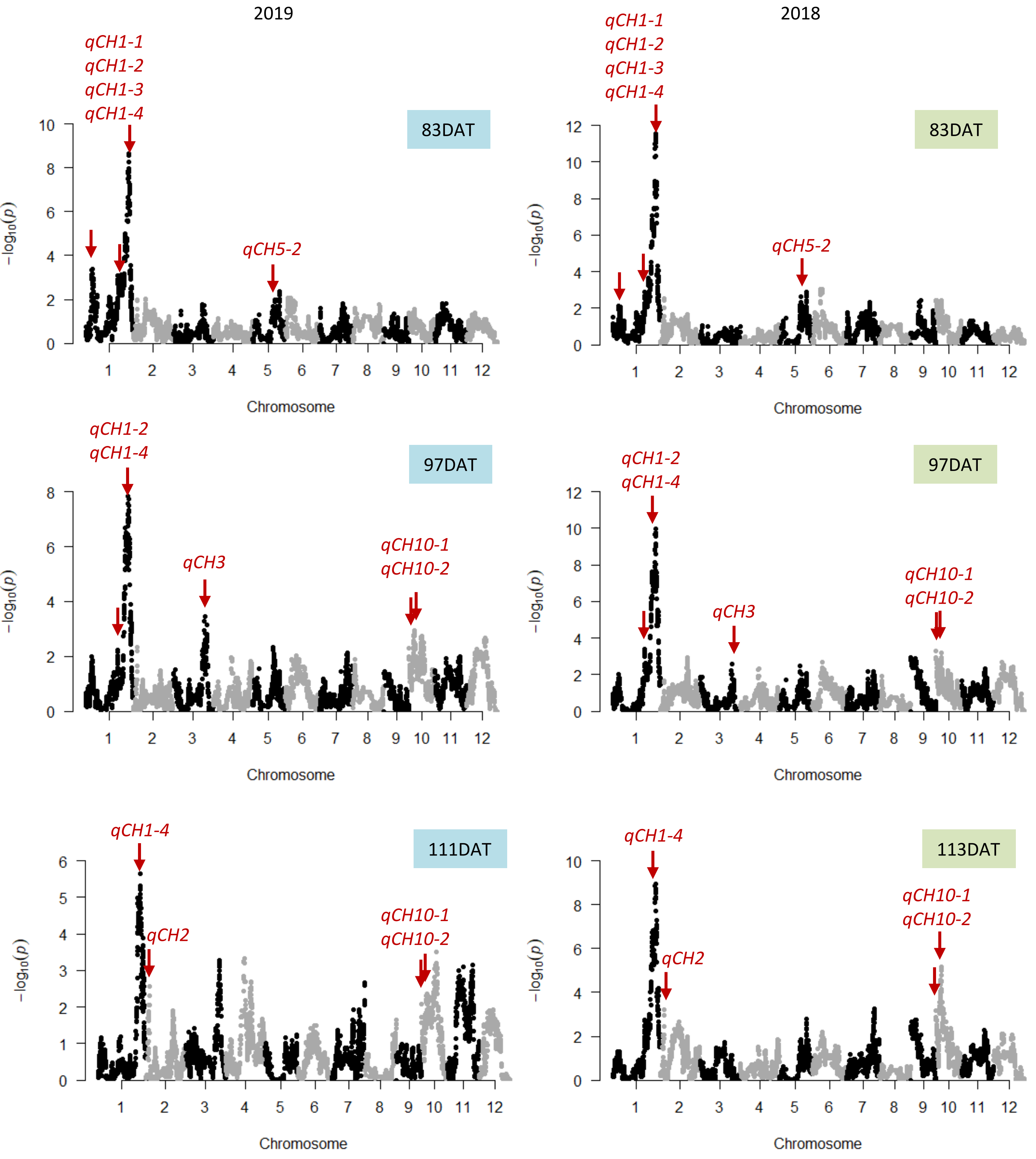

Supplementary Figure 5. GWAS of CH using JAM lines.
Continued.

A

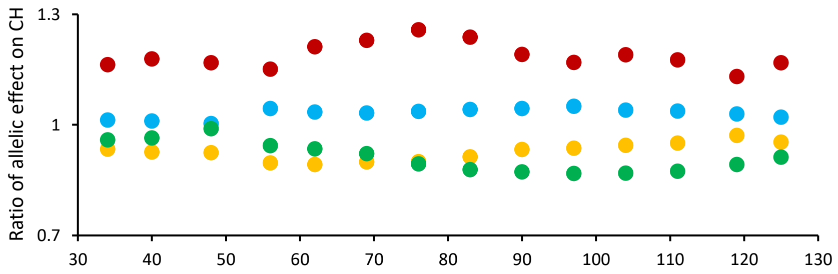

	DAT (2019)													
Sd1 allele	34	40	48	56	62	69	76	83	90	97	104	111	119	125
A	c	b	b	c	c	c	c	c	c	c	c	c	c	c
B	b	a	a	b	b	b	b	b	b	b	b	b	bc	b
C	a	a	a	a	a	a	a	a	a	a	a	a	ab	ab
D	ab	a	a	a	a	a	a	a	a	a	a	a	a	a

B

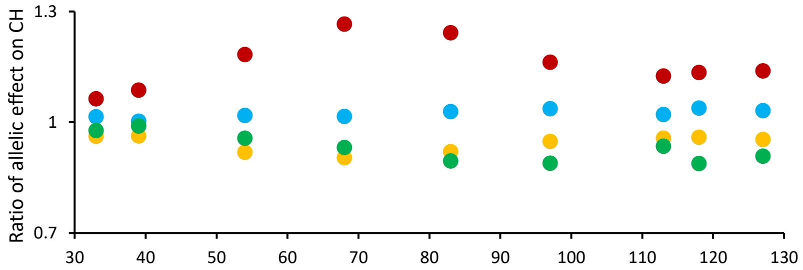

	DAT (2018)								
Sd1 allele	33	39	54	68	83	97	113	118	127
A	a	a	c	c	c	c	c	b	c
B	a	a	b	b	b	b	b	b	b
C	a	a	a	a	a	a	ab	a	ab
D	a	a	ab	a	a	a	a	a	a

Supplementary Figure 6. Statistical analysis of allelic effect of *Sd1* on CH.
(A,B) Time-course pattern of four *Sd1* allelic effects on CH at *qCH1-4* is shown in the bottom of Figure 2B in 2019 (A) and in 2018 (B). The Tukey-Kramer test was performed among the four *Sd1* alleles at each DAT. Different lower case letters indicate significant differences (P<0.05). Time points where the categories were differentiated (A, B and C,D) are shown in red.

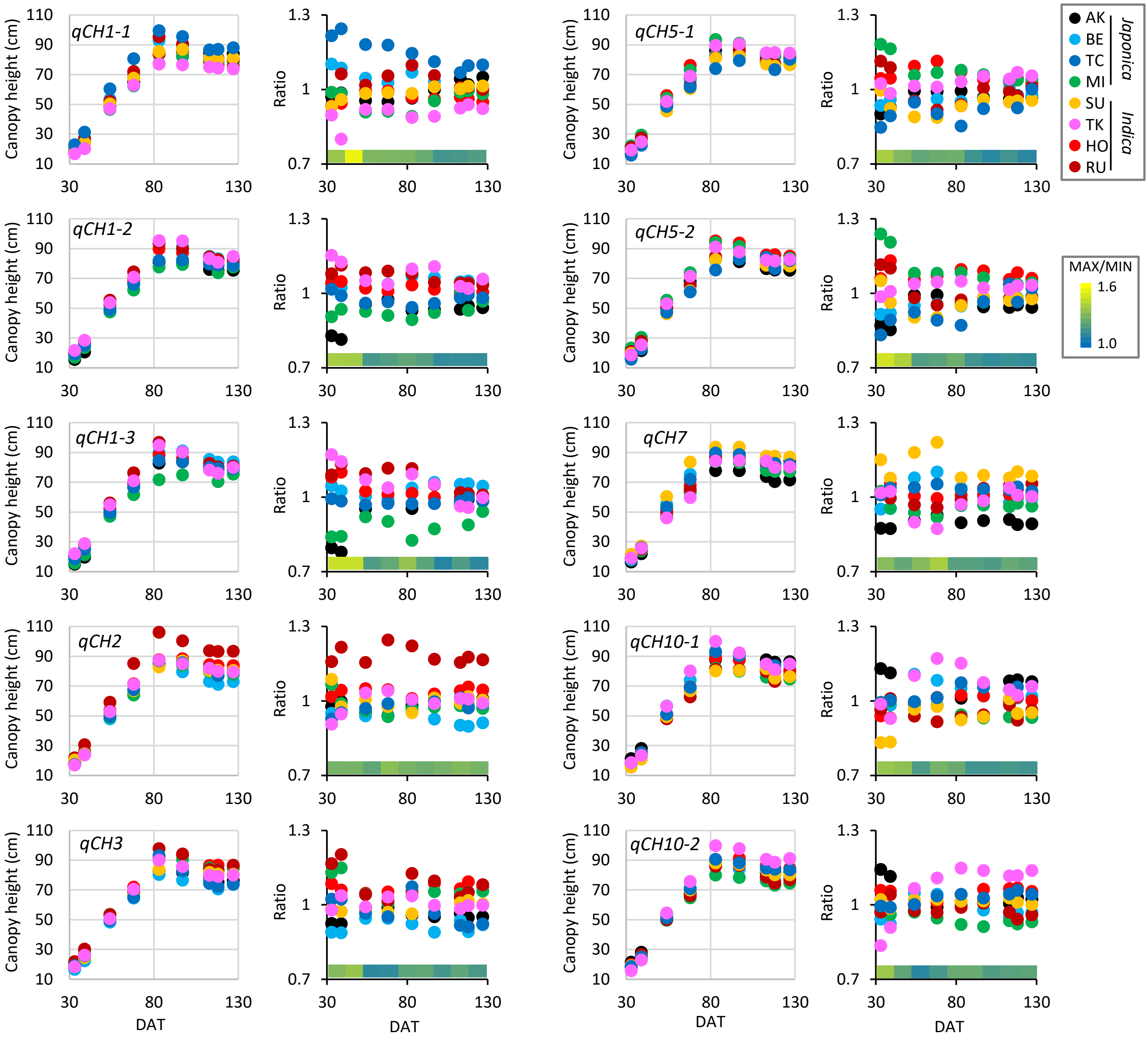

Supplementary Figure 7. Time-course pattern of haplotype effects on CH at *qCH* QTLs except for *qCH1-4*. Average of haplotype effect on CH (left) and ratio of the haplotype effect to averaged values in the JAM lines (right) at each DAT in 2018 were plotted.

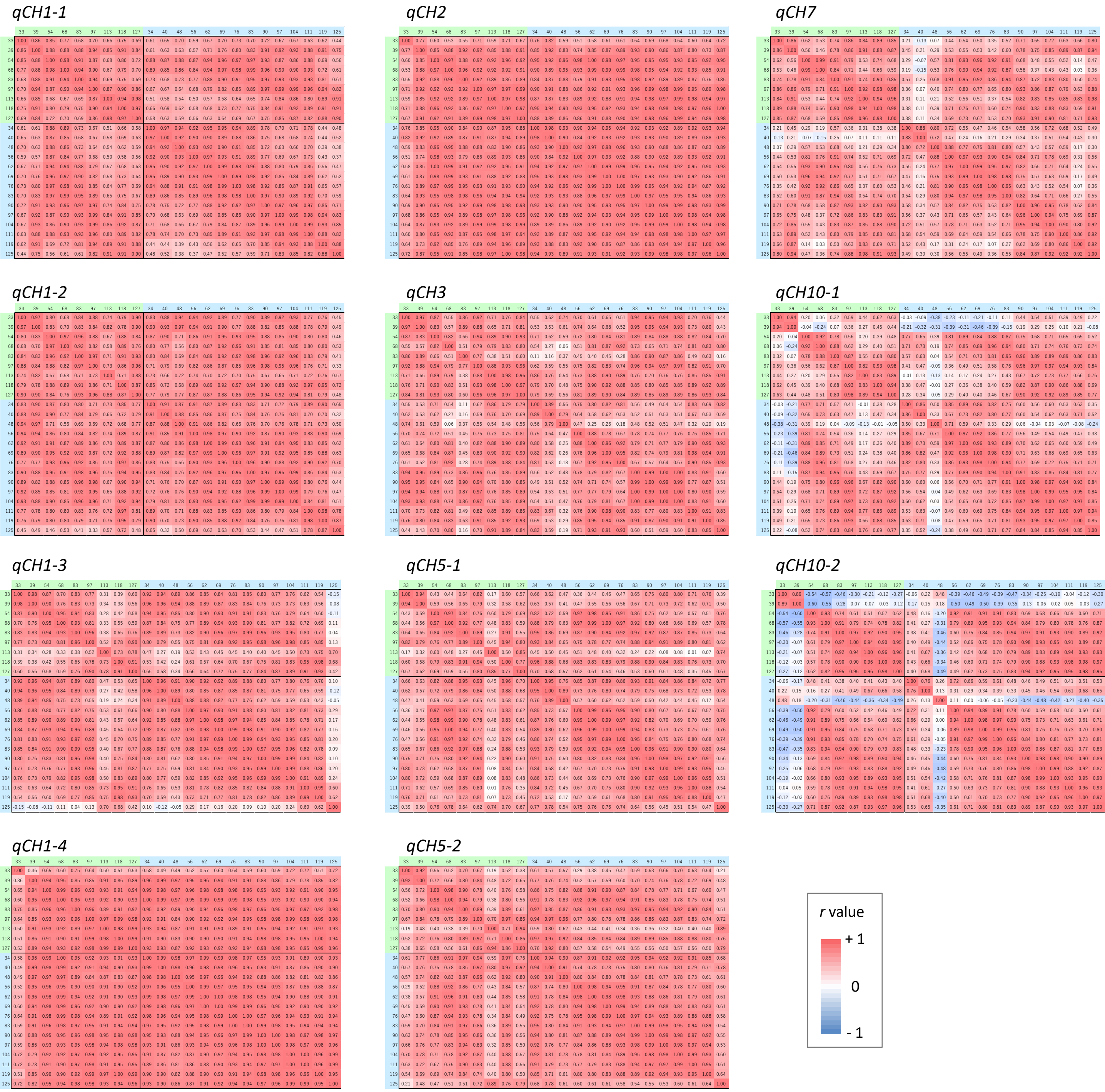

Supplementary Figure 8. Pearson correlations among haplotype effects on CH in 2019 and 2018 at *qCH* QTLs. The Pearson coefficient r values were examined using data of haplotype effect on CH shown in Supplementary Data 4. There are 9 and 14 DAT time points in 2018 (green background) and 2019 (blue background), respectively. Heat maps of r values are shown.

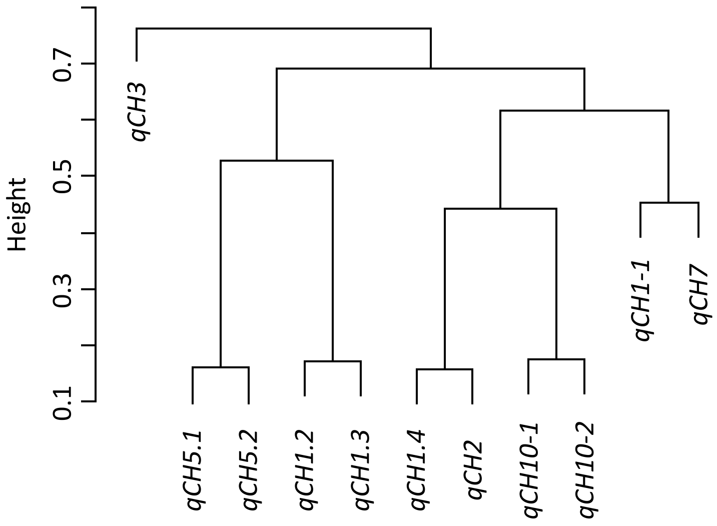

Supplementary Figure 9. Clustering analysis of *qCH* QTLs.
Hierarchical clustering was carried out using data in Supplementary Figure 8. Height indicates the distance between QTLs.

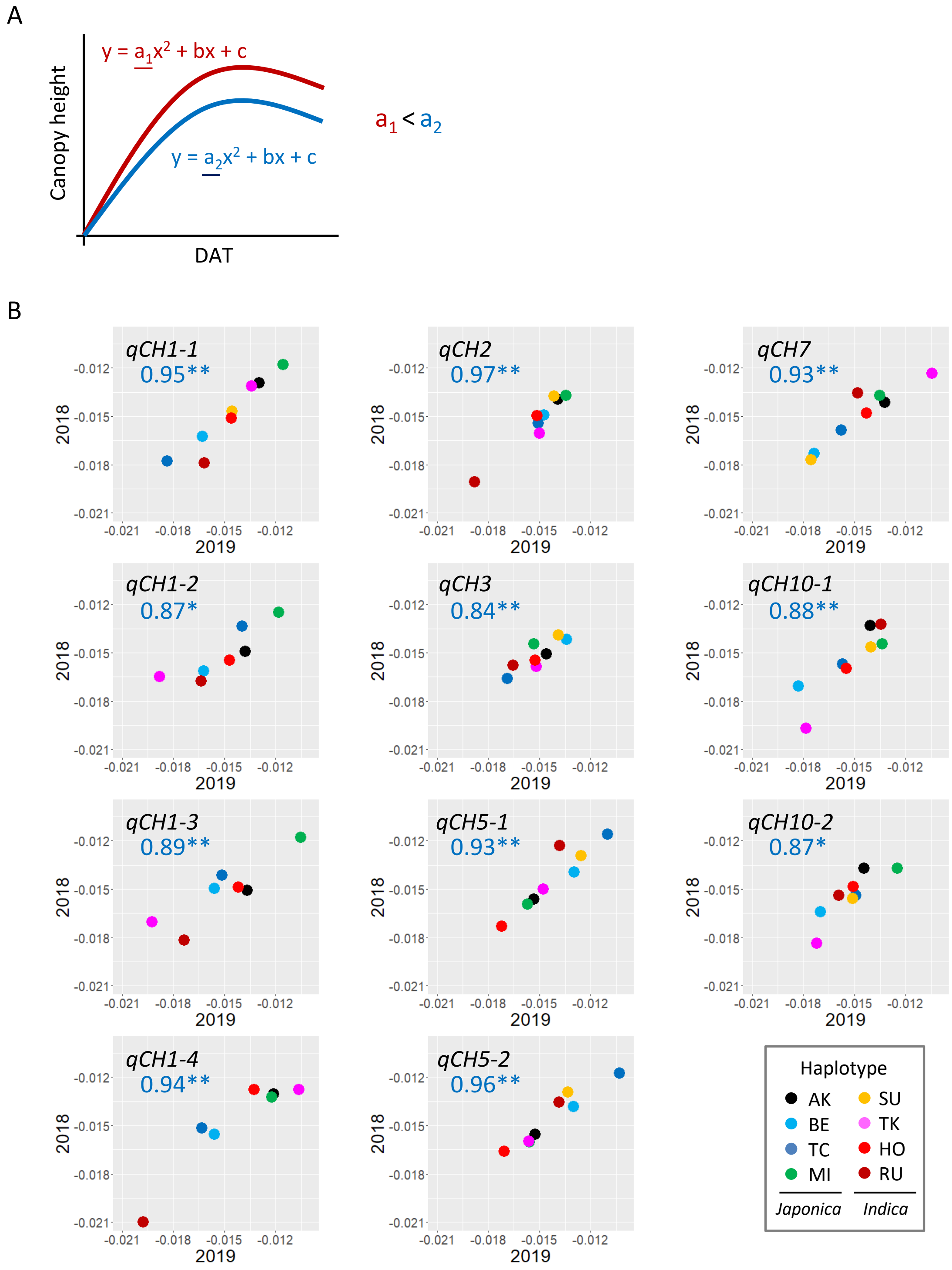

Supplementary Figure 10. Plot of haplotype effect on coefficient "a" at *qCH* QTLs in 2019 and 2018. (A) Time-course patterns of haplotype effects on CH in 2019 and 2018 were applied to the model ($y = ax^2 + bx + c$). (B) Coefficient "a" data of the model in 2019 and 2018 were plotted. Pearson correlation *r* values between the two years were shown in blue. Asterisks indicate significant correlations (Pairwise two-sided, **P<0.01, *P<0.05).

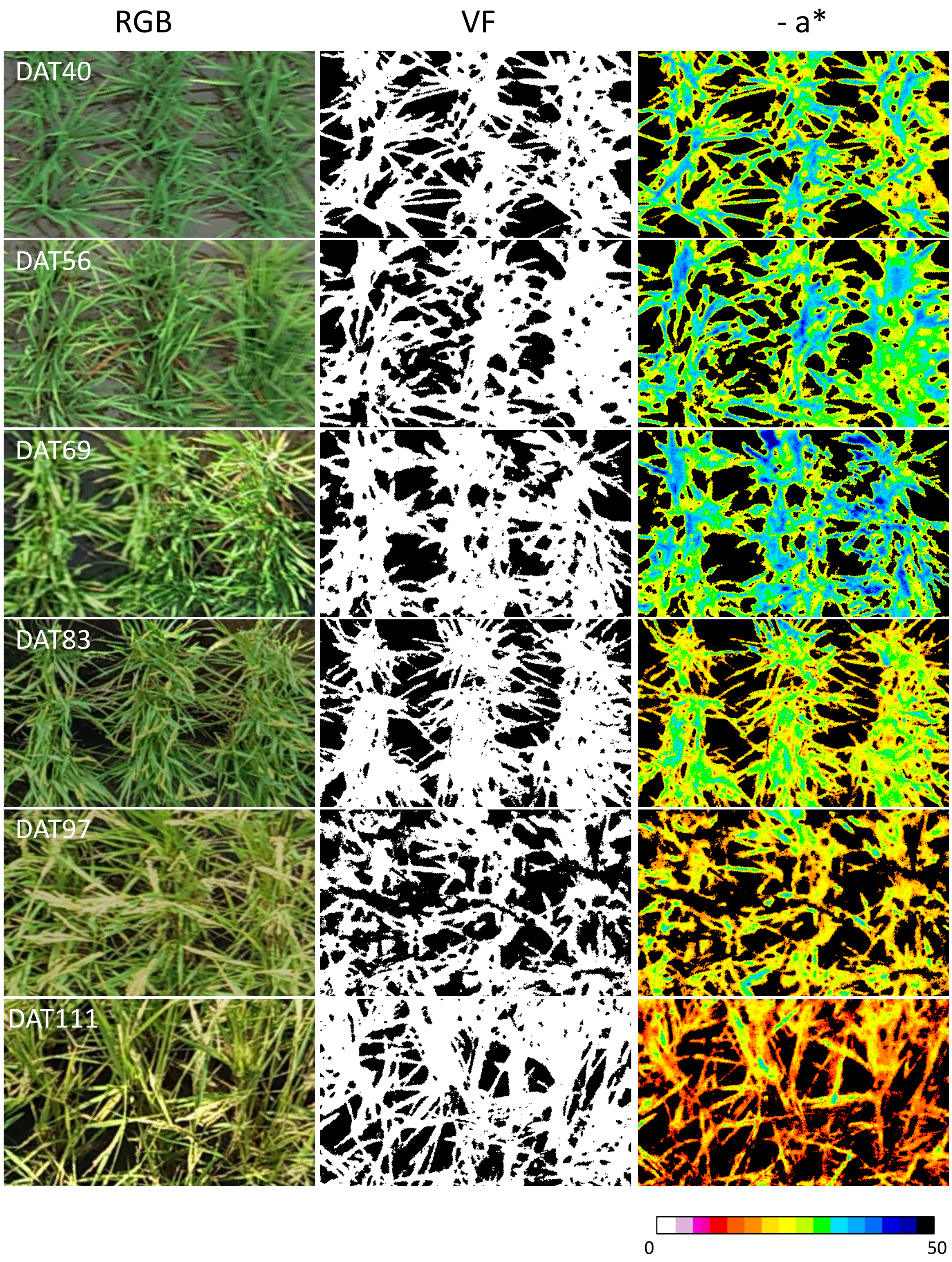

Supplementary Figure 11. Change of -a* data in the process of development.
RGB (left), VF (middle) and -a*(right) images of a JAM line from 40 to 111 DAT were aligned.

A

	Year	
	2019	2018
qCH1-1	0.72	0.82
qCH1-2	0.88	0.89
qCH1-3	0.92	0.88
qCH1-4	0.59	0.39
qCH2	0.66	0.65
qCH3	0.08	0.70
qCH5-1	0.24	0.82
qCH5-2	0.49	0.83
qCH7	0.68	0.67
qCH10-1	0.06	0.85
qCH10-2	-0.10	0.78

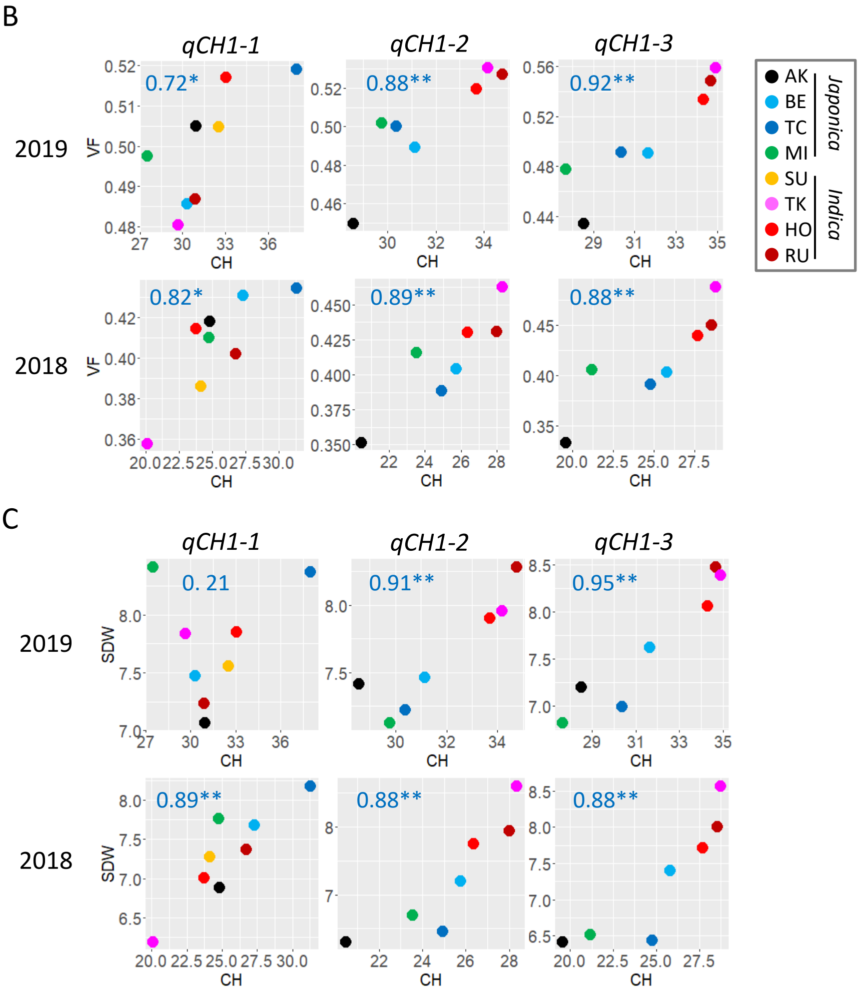

Supplementary Figure 12. Relationship between CH and VF in the JAM lines.
(A) Pearson correlation r values between CH and VF at *qCH* QTLs in 2019[40DAT] and 2018[39DAT] were shown. Underlines indicate significant correlations (Pairwise two-sided, *P<0.05). (B,C) Plots of haplotype effect between CH and VF (B) or SDW (C) in 2019 (top) and 2018 (bottom) at *qCH1-1*, *qCH1-2* and *qCH1-3*. Numbers in blue indicate Pearson's r. Asterisks indicate significant correlations (Pairwise two-sided, **P<0.01, *P<0.05).

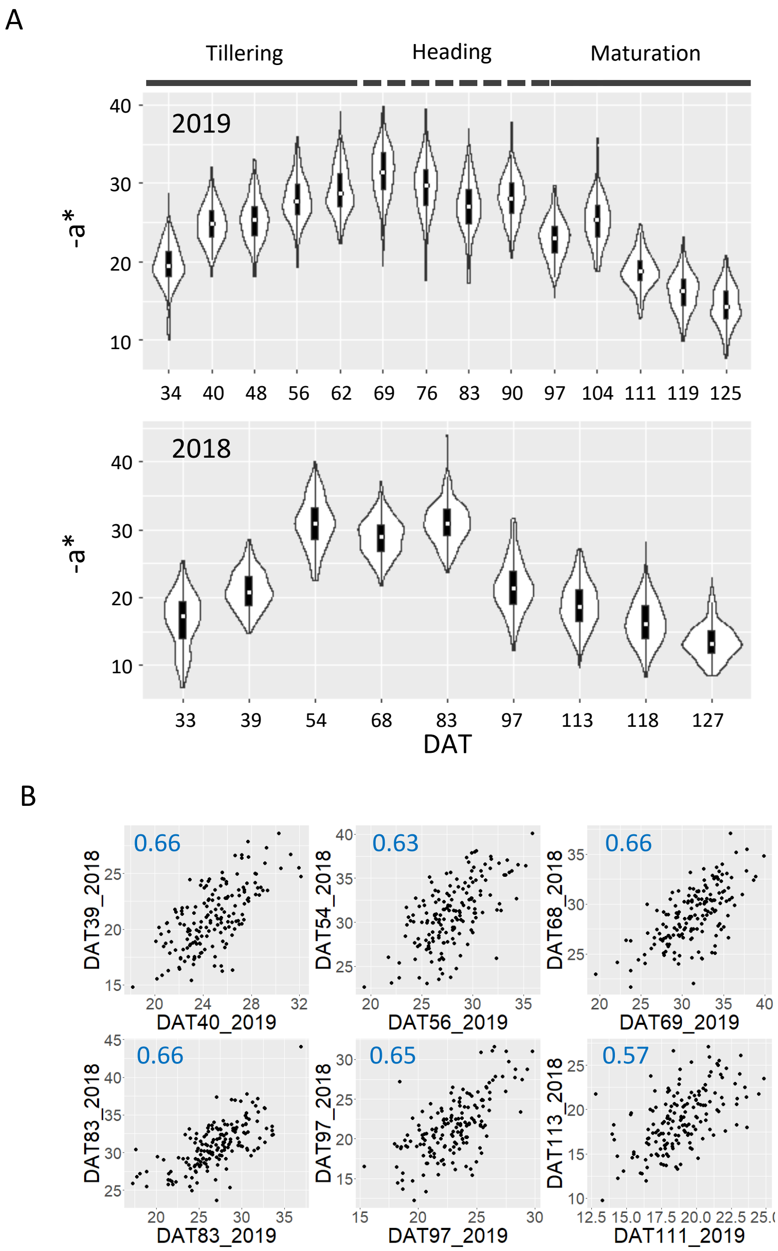

Supplementary Figure 13. Features of –a* data in JAM lines.
(A) Violin plots of –a* data of the 165 JAM lines in 2019 (top) and 2018 (bottom). (B) Relationship between data of –a* in 2019 and 2018 at the selected six points of DATs. Numbers in blue indicate Pearson's *r*.

	r value (CH & -a* [2019])						*r* value (CH & -a* [2018])					
	40	56	69	83	97	111	39	54	68	83	97	113
JAM	0.49	0.25	0.06	0.29	0.48	0.10	0.76	0.20	0.03	0.38	0.35	0.27
qCH1-1	0.71	0.76	0.39	0.44	0.69	-0.01	0.86	0.67	0.35	0.76	0.25	0.38
qCH1-2	0.83	0.90	0.68	0.74	0.87	0.13	0.94	0.69	0.33	0.75	0.39	-0.34
qCH1-3	0.91	0.80	0.80	0.77	0.78	0.13	0.95	0.82	0.72	0.77	-0.02	-0.29
qCH1-4	0.77	0.92	0.74	0.57	0.62	-0.62	0.30	0.72	0.86	0.81	-0.11	-0.33
qCH2	0.80	0.55	0.05	0.29	0.05	-0.40	0.84	0.47	0.11	0.71	-0.52	-0.19
qCH3	0.28	0.66	0.67	0.85	0.94	0.53	0.95	0.74	0.44	0.74	0.89	0.70
qCH5-1	-0.24	0.18	-0.33	0.50	0.85	0.20	0.59	0.33	0.29	0.27	-0.06	0.36
qCH5-2	0.52	0.66	0.26	0.75	0.78	0.47	0.85	0.53	0.80	0.57	-0.30	0.15
qCH7	0.95	0.69	0.44	0.69	0.78	0.40	0.65	-0.11	-0.26	0.94	0.82	0.94
qCH10-1	0.21	0.40	0.16	0.66	0.75	0.45	0.89	0.04	0.42	0.28	0.55	0.72
qCH10-2	-0.09	-0.26	-0.50	0.33	0.28	-0.16	0.84	-0.53	0.21	-0.17	-0.06	0.23

Supplementary Figure 14. Relationship between CH and –a* data at *qCH* QTLs.
Pearson correlation r values between CH and –a* at *qCH* in 2019 and 2018 were shown. As a control, the correlations between CH and –a* data at the JAM line level are shown in the first row.

A

2019

JAM	DAT					
Trait	40	56	69	83	97	111
CL	0.45	0.65	0.77	0.87	0.84	0.73
PL	0.29	0.29	0.35	0.41	0.57	0.56
PN	-0.18	-0.31	-0.45	-0.39	-0.42	-0.38
PW	0.07	0.03	-0.02	0.09	0.18	0.01
SLW	0.28	0.30	0.39	0.43	0.50	0.51
TW	0.26	0.25	0.29	0.38	0.48	0.40

2018

JAM	DAT					
Trait	39	54	68	83	97	113
CL	0.35	0.58	0.62	0.78	0.81	0.56
PL	0.07	0.25	0.25	0.20	0.29	0.20
PN	-0.04	-0.15	-0.19	-0.23	-0.35	-0.25
PW	0.12	-0.15	-0.21	-0.08	-0.07	-0.16
SLW	0.37	0.30	0.22	0.33	0.44	0.36
TW	0.37	0.17	0.06	0.23	0.33	0.21

B

2019

qCH1-1	DAT					
Trait	40	56	69	83	97	111
CL	0.77	0.85	0.94	0.96	0.94	0.96
PL	0.81	0.78	0.92	0.86	0.80	0.84
PN	-0.78	-0.79	-0.82	-0.69	-0.52	-0.61
PW	-0.03	-0.13	-0.16	-0.05	0.19	0.07
SLW	0.27	0.39	0.53	0.54	0.76	0.65
TW	0.22	0.28	0.39	0.44	0.70	0.57

2018

qCH1-1	DAT					
Trait	39	54	68	83	97	113
CL	0.85	0.89	0.93	0.98	0.95	0.76
PL	0.66	0.82	0.85	0.66	0.64	0.46
PN	-0.08	-0.45	-0.44	-0.35	-0.23	-0.17
PW	0.20	0.09	0.10	0.12	0.33	0.48
SLW	0.79	0.79	0.72	0.75	0.76	0.60
TW	0.77	0.71	0.66	0.69	0.81	0.75

2019

qCH5-1	DAT					
Trait	40	56	69	83	97	111
CL	0.73	0.78	0.83	0.96	0.98	0.92
PL	0.68	0.77	0.73	0.80	0.81	0.63
PN	-0.36	-0.61	-0.53	-0.34	-0.12	-0.03
PW	0.33	0.11	0.23	0.45	0.61	0.63
SLW	0.53	0.71	0.74	0.83	0.87	0.71
TW	0.50	0.41	0.52	0.73	0.88	0.82

2018

qCH5-1	DAT					
Trait	39	54	68	83	97	113
CL	0.72	0.76	0.83	0.96	0.92	0.16
PL	0.67	0.56	0.48	0.41	0.60	0.75
PN	0.42	0.01	0.11	0.23	0.09	-0.51
PW	-0.19	-0.40	-0.24	0.00	-0.16	-0.67
SLW	0.84	0.44	0.33	0.29	0.45	0.42
TW	0.73	0.10	0.14	0.31	0.34	-0.16

qCH1-2	DAT					
Trait	40	56	69	83	97	111
CL	0.77	0.91	0.93	0.95	0.99	0.75
PL	0.51	0.63	0.56	0.46	0.39	0.79
PN	0.16	-0.35	-0.31	-0.27	-0.32	-0.46
PW	0.50	0.55	0.54	0.57	0.72	0.37
SLW	0.79	0.68	0.66	0.62	0.49	0.53
TW	0.95	0.88	0.86	0.85	0.83	0.66

qCH1-2	DAT					
Trait	39	54	68	83	97	113
CL	0.87	0.88	0.82	0.96	0.99	0.72
PL	0.17	0.23	0.29	0.14	0.00	-0.15
PN	0.77	0.41	0.19	0.48	0.55	0.68
PW	0.23	-0.16	-0.32	-0.02	0.10	-0.10
SLW	0.85	0.66	0.49	0.69	0.77	0.65
TW	0.76	0.43	0.23	0.52	0.64	0.45

qCH5-2	DAT					
Trait	40	56	69	83	97	111
CL	0.74	0.89	0.89	0.98	0.98	0.92
PL	0.75	0.78	0.80	0.81	0.79	0.80
PN	-0.71	-0.62	-0.64	-0.44	-0.31	-0.32
PW	0.08	0.21	0.33	0.50	0.54	0.49
SLW	0.66	0.80	0.80	0.88	0.93	0.90
TW	0.43	0.60	0.67	0.83	0.88	0.83

qCH5-2	DAT					
Trait	39	54	68	83	97	113
CL	0.77	0.88	0.92	0.96	0.88	0.41
PL	0.63	0.48	0.46	0.46	0.52	0.59
PN	0.30	0.01	-0.01	0.08	-0.08	-0.50
PW	0.04	-0.25	-0.17	0.02	0.09	-0.23
SLW	0.75	0.69	0.58	0.51	0.59	0.58
TW	0.76	0.53	0.47	0.51	0.64	0.43

qCH1-3	DAT					
Trait	40	56	69	83	97	111
CL	0.80	0.87	0.94	0.97	0.95	0.77
PL	0.63	0.65	0.69	0.76	0.88	0.90
PN	-0.31	-0.51	-0.58	-0.68	-0.85	-0.73
PW	0.46	0.42	0.50	0.63	0.70	0.32
SLW	0.71	0.75	0.64	0.53	0.37	0.53
TW	0.92	0.92	0.89	0.88	0.79	0.67

qCH1-3	DAT					
Trait	39	54	68	83	97	113
CL	0.88	0.91	0.84	0.97	0.94	0.35
PL	0.49	0.50	0.53	0.69	0.70	0.08
PN	0.10	-0.12	-0.19	-0.38	-0.50	0.12
PW	0.21	-0.08	-0.33	-0.08	-0.08	-0.47
SLW	0.88	0.93	0.80	0.92	0.85	0.16
TW	0.74	0.60	0.37	0.60	0.55	-0.17

qCH7	DAT					
Trait	40	56	69	83	97	111
CL	0.55	0.99	0.90	0.93	0.74	0.76
PL	0.01	0.70	0.71	0.72	0.85	0.69
PN	0.09	-0.71	-0.80	-0.77	-0.70	-0.69
PW	-0.50	0.11	0.20	0.14	0.19	-0.34
SLW	0.57	0.26	0.10	0.16	0.25	0.13
TW	0.09	0.33	0.26	0.26	0.39	-0.17

qCH7	DAT					
Trait	39	54	68	83	97	113
CL	0.68	0.91	0.86	0.94	0.89	0.69
PL	0.60	0.80	0.73	0.83	0.82	0.57
PN	-0.50	-0.84	-0.80	-0.76	-0.70	-0.40
PW	-0.68	-0.50	-0.45	-0.71	-0.66	-0.71
SLW	0.78	0.34	0.25	0.66	0.75	0.85
TW	0.27	-0.22	-0.32	-0.01	0.26	0.34

qCH1-4	DAT					
Trait	40	56	69	83	97	111
CL	0.94	0.97	0.97	0.99	0.98	0.94
PL	0.72	0.79	0.79	0.85	0.92	0.95
PN	-0.80	-0.86	-0.83	-0.87	-0.89	-0.76
PW	-0.85	-0.74	-0.73	-0.72	-0.68	-0.58
SLW	0.95	0.97	0.93	0.88	0.85	0.78
TW	0.66	0.79	0.73	0.68	0.66	0.64

qCH1-4	DAT					
Trait	39	54	68	83	97	113
CL	0.93	0.96	0.97	0.94	0.95	0.93
PL	0.23	0.28	0.30	0.41	0.54	0.43
PN	-0.83	-0.83	-0.82	-0.80	-0.90	-0.90
PW	-0.77	-0.92	-0.91	-0.95	-0.91	-0.85
SLW	0.67	0.80	0.76	0.79	0.85	0.87
TW	0.16	0.20	0.13	0.12	0.32	0.45

qCH10-1	DAT					
Trait	40	56	69	83	97	111
CL	0.68	0.68	0.82	0.94	0.95	0.91
PL	0.56	0.45	0.58	0.77	0.98	0.91
PN	-0.61	-0.66	-0.72	-0.54	-0.45	-0.45
PW	-0.47	-0.52	-0.70	-0.66	-0.31	-0.34
SLW	0.51	0.44	0.63	0.85	0.92	0.92
TW	0.32	0.22	0.34	0.60	0.87	0.85

qCH10-1	DAT					
Trait	39	54	68	83	97	113
CL	0.25	0.64	0.70	0.87	0.86	0.49
PL	0.49	0.15	-0.03	0.19	0.32	-0.01
PN	0.12	0.37	0.03	-0.17	-0.11	-0.15
PW	0.36	-0.69	-0.82	-0.67	-0.50	-0.50
SLW	0.60	0.29	0.21	0.49	0.67	0.31
TW	0.62	-0.20	-0.34	-0.05	0.17	-0.08

qCH2	DAT					
Trait	40	56	69	83	97	111
CL	0.91	0.90	0.98	0.98	0.95	0.92
PL	0.90	0.94	0.88	0.81	0.87	0.89
PN	-0.51	-0.68	-0.46	-0.39	-0.61	-0.62
PW	-0.56	-0.65	-0.57	-0.53	-0.67	-0.67
SLW	0.71	0.72	0.86	0.88	0.78	0.73
TW	0.38	0.33	0.52	0.57	0.38	0.32

qCH2	DAT					
Trait	39	54	68	83	97	113
CL	0.85	0.93	0.93	0.87	0.90	0.86
PL	0.81	0.85	0.82	0.75	0.79	0.75
PN	-0.35	-0.49	-0.34	-0.21	-0.47	-0.58
PW	-0.61	-0.86	-0.90	-0.82	-0.70	-0.76
SLW	0.88	0.75	0.78	0.82	0.88	0.76
TW	0.34	-0.05	-0.06	0.07	0.26	0.07

qCH10-2	DAT					
Trait	40	56	69	83	97	111
CL	0.40	0.70	0.87	0.95	0.98	0.87
PL	0.48	0.62	0.77	0.74	0.86	0.85
PN	-0.54	-0.71	-0.76	-0.64	-0.33	-0.53
PW	-0.54	-0.57	-0.73	-0.75	-0.48	-0.59
SLW	0.14	0.15	0.41	0.56	0.83	0.68
TW	-0.02	-0.01	0.20	0.34	0.68	0.50

qCH10-2	DAT					
Trait	39	54	68	83	97	113
CL	-0.10	0.67	0.81	0.96	0.99	0.88
PL	-0.19	0.11	0.14	-0.04	-0.17	-0.17
PN	0.03	-0.17	-0.46	-0.69	-0.71	-0.88
PW	0.12	-0.75	-0.84	-0.91	-0.90	-0.84
SLW	-0.22	0.59	0.77	0.90	0.88	0.77
TW	-0.24	-0.13	0.07	0.21	0.19	0.06

qCH3	DAT					
Trait	40	56	69	83	97	111
CL	0.59	0.80	0.80	0.99	0.99	0.81
PL	0.70	0.59	0.86	0.66	0.66	0.85
PN	-0.82	-0.55	-0.60	-0.31	-0.31	-0.59
PW	-0.17	-0.19	-0.29	0.25	0.28	-0.18
SLW	0.68	0.73	0.78	0.85	0.85	0.82
TW	0.23	0.24	0.19	0.65	0.67	0.30

qCH3	DAT					
Trait	39	54	68	83	97	113
CL	0.96	0.91	0.71	0.88	0.97	0.77
PL	0.47	0.69	0.61	0.06	0.61	0.87
PN	-0.56	-0.48	-0.13	-0.36	-0.58	-0.55
PW	-0.39	-0.08	-0.35	-0.46	-0.27	-0.08
SLW	0.55	0.61	0.46	0.45	0.69	0.60
TW	0.34	0.55	0.27	0.21	0.53	0.54

Supplementary Figure 15. Relationships between CH at each DAT and yield traits in 2019 and 2018. (A) Pearson correlation *r* values between CH at each DAT and the traits in 165 JAM lines are shown. (B) Pearson correlation *r* values of haplotype effects between CH at each DAT and the traits in 165 JAM lines are shown. Underlines indicate significant correlations (Pairwise two-sided, P<0.05).

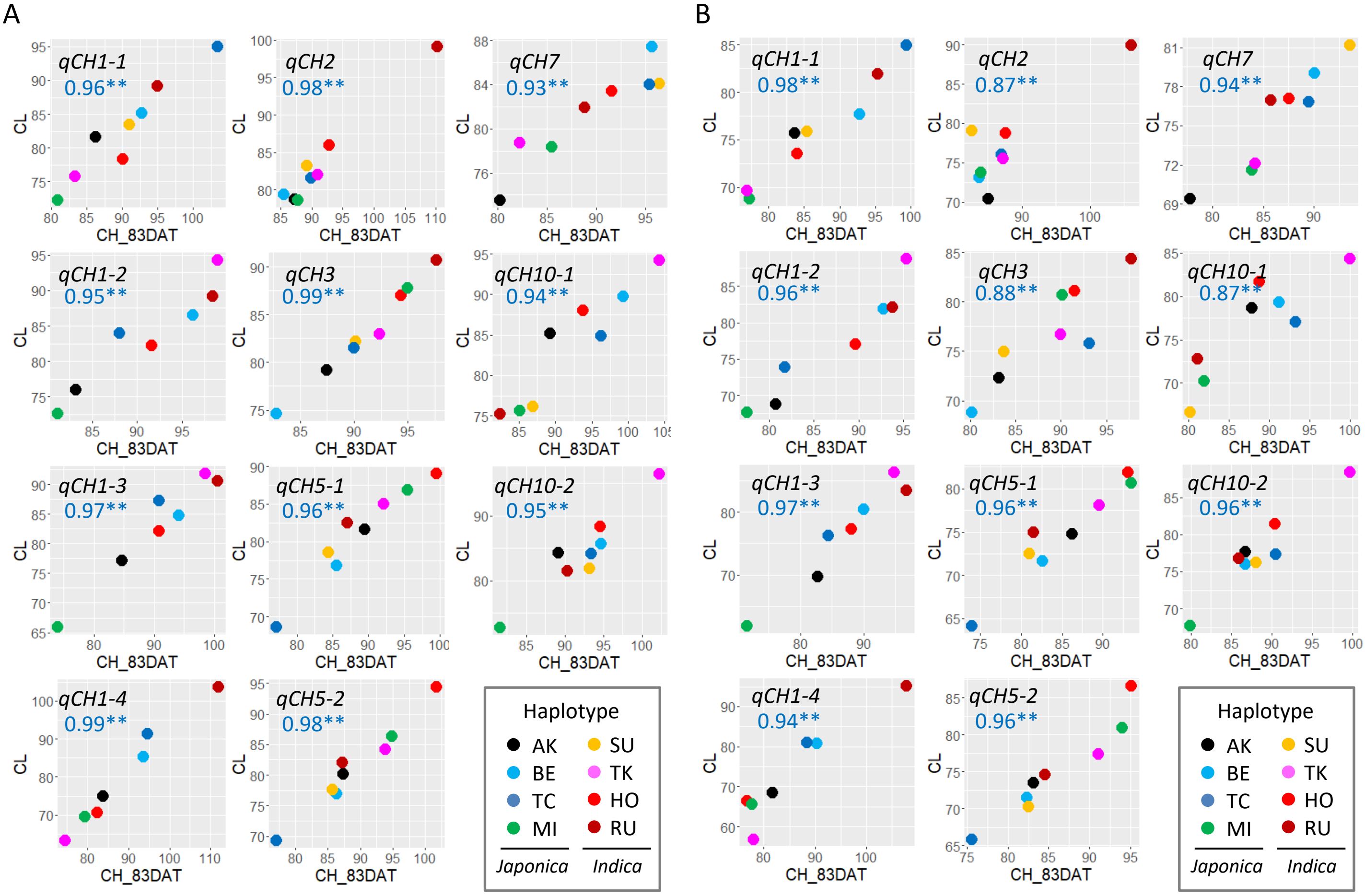

Supplementary Figure 16. Relationships between CH and CL at *qCH* QTLs.
Haplotype effects on CH at 83DAT and CL in 2019 (A) and 2018 (B) were plotted. Numbers in blue indicate Pearson's *r*. Asterisks indicate significant correlations (Pairwise two-sided, **P<0.01).

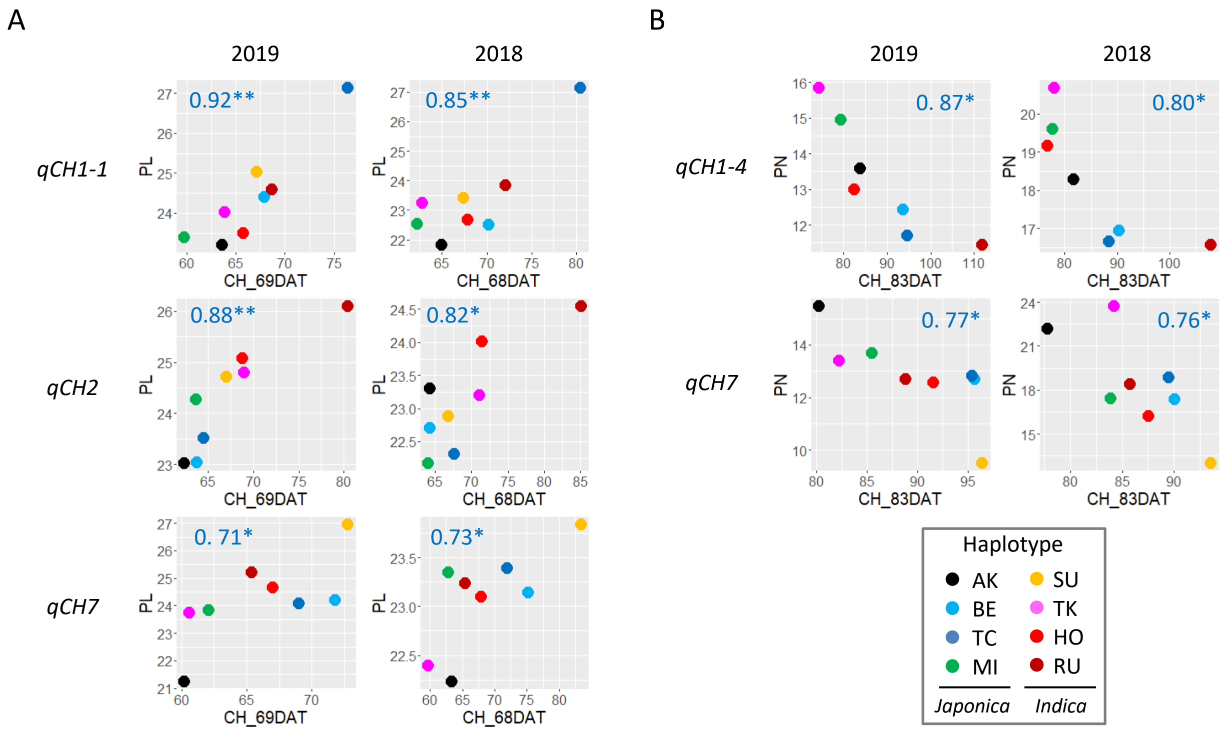

Supplementary Figure 17. Relationships between CH and PL or PN at *qCH* QTLs.
(A) Haplotype effects on PL and CH at 69 DAT in 2019 or CH at 68 DAT in 2018 at *qCH1-1*, *qCH2* and *qCH7* were plotted. (B) Haplotype effects on PN and CH at 69 DAT in 2019 or CH at 68 DAT in 2018 at *qCH1-4* and *qCH7* were plotted. Numbers in blue indicate Pearson's *r*. Asterisks indicate significant correlations (Pairwise two-sided, **P<0.01, *P<0.05).
